# Characterizing the onset and progression of Alzheimer’s pathologies using amyloid and tau PET imaging and plasma p-tau217

**DOI:** 10.1093/braincomms/fcaf449

**Published:** 2025-11-18

**Authors:** Karly A Cody, Rebecca E Langhough, Shorena Janelidze, Erin M Jonaitis, Rachael E Wilson, Bradley T Christian, Sanjay Asthana, Sterling C Johnson, Henrik Zetterberg, Oskar Hansson, Tobey J Betthauser

**Affiliations:** Wisconsin Alzheimer’s Disease Research Center, University of Wisconsin School of Medicine and Public Health, Madison, WI 53792, USA; Department of Medicine, University of Wisconsin-Madison School of Medicine and Public Health, Madison, WI 53705, USA; Department of Neurology and Neurological Sciences, Stanford University, Palo Alto, CA 94305, USA; Wisconsin Alzheimer’s Disease Research Center, University of Wisconsin School of Medicine and Public Health, Madison, WI 53792, USA; Department of Medicine, University of Wisconsin-Madison School of Medicine and Public Health, Madison, WI 53705, USA; Wisconsin Alzheimer’s Institute, University of Wisconsin School of Medicine and Public Health, Madison, WI 53726, USA; Clinical Memory Research Unit, Department of Clinical Sciences, Malmö, Lund University, Lund 205 02, Sweden; Wisconsin Alzheimer’s Disease Research Center, University of Wisconsin School of Medicine and Public Health, Madison, WI 53792, USA; Department of Medicine, University of Wisconsin-Madison School of Medicine and Public Health, Madison, WI 53705, USA; Wisconsin Alzheimer’s Institute, University of Wisconsin School of Medicine and Public Health, Madison, WI 53726, USA; Wisconsin Alzheimer’s Disease Research Center, University of Wisconsin School of Medicine and Public Health, Madison, WI 53792, USA; Department of Medicine, University of Wisconsin-Madison School of Medicine and Public Health, Madison, WI 53705, USA; Wisconsin Alzheimer’s Institute, University of Wisconsin School of Medicine and Public Health, Madison, WI 53726, USA; Wisconsin Alzheimer’s Disease Research Center, University of Wisconsin School of Medicine and Public Health, Madison, WI 53792, USA; Waisman Laboratory for Brain Imaging and Behavior, University of Wisconsin-Madison, Madison, WI 53705, USA; Department of Medical Physics, University of Wisconsin-Madison School of Medicine and Public Health, Madison, WI 53705, USA; Wisconsin Alzheimer’s Disease Research Center, University of Wisconsin School of Medicine and Public Health, Madison, WI 53792, USA; Department of Medicine, University of Wisconsin-Madison School of Medicine and Public Health, Madison, WI 53705, USA; Wisconsin Alzheimer’s Disease Research Center, University of Wisconsin School of Medicine and Public Health, Madison, WI 53792, USA; Department of Medicine, University of Wisconsin-Madison School of Medicine and Public Health, Madison, WI 53705, USA; Wisconsin Alzheimer’s Institute, University of Wisconsin School of Medicine and Public Health, Madison, WI 53726, USA; Wisconsin Alzheimer’s Disease Research Center, University of Wisconsin School of Medicine and Public Health, Madison, WI 53792, USA; Department of Psychiatry and Neurochemistry, Institute of Neuroscience and Physiology, the Sahlgrenska Academy at the University of Gothenburg, Mölndal 405 30, Sweden; Clinical Neurochemistry Laboratory, Sahlgrenska University Hospital, Mölndal 413 45, Sweden; Department of Neurodegenerative Disease, University College London Institute of Neurology, London WC1N 3BG, UK; UK Dementia Research Institute at University College London, London NW1 3BT, UK; Hong Kong Center for Neurodegenerative Diseases, Clear Water Bay, Hong Kong, China; Clinical Memory Research Unit, Department of Clinical Sciences, Malmö, Lund University, Lund 205 02, Sweden; Memory Clinic, Skåne University Hospital, Malmö 205 01, Sweden; Wisconsin Alzheimer’s Disease Research Center, University of Wisconsin School of Medicine and Public Health, Madison, WI 53792, USA; Department of Medicine, University of Wisconsin-Madison School of Medicine and Public Health, Madison, WI 53705, USA; Department of Medical Physics, University of Wisconsin-Madison School of Medicine and Public Health, Madison, WI 53705, USA

**Keywords:** amyloid, tau, Alzheimer’s, timing, p-tau217

## Abstract

Characterizing the onset and progression of Alzheimer’s disease pathologies relative to one another is important for biological staging and clinical trial design. Recent advances in blood plasma assays of Alzheimer’s disease amyloid and tau pathology have enabled detection of Alzheimer’s disease pathophysiology during life, but it remains unclear when plasma biomarker abnormalities are detectable relative to established amyloid and tau PET imaging biomarkers, and the extent to which plasma biomarkers can be used for biological disease staging. This work applies a novel temporal modelling approach to amyloid PET and plasma p-tau217 data from two different assay platforms to characterize when plasma p-tau217 become abnormal relative to amyloid PET, tau PET, and cognitive decline in a predominantly cognitively unimpaired cohort.

This study included a subset of 172 Wisconsin Registry for Alzheimer’s Prevention participants (mean (standard deviation (SD)) age at baseline plasma = 63.2 (6.3) years; 149 cognitively unimpaired at last cognitive assessment) with available amyloid PET imaging and plasma p-tau217 data assayed on the Lilly Meso Scale Delivery and Quanterix Alzpath platforms. We estimated the within-person onsets of detectable amyloid PET and plasma p-tau217 using sampled iterative local approximation and investigated the impact of this timing on downstream tau PET accumulation and cognitive decline using linear mixed-effects models.

Longitudinal modelling revealed that on average, amyloid PET positivity preceded p-tau217 positivity, and both amyloid and p-tau217 preceded detectable changes in brain tau accumulation. Comparisons of ‘time from biomarker onset’ indicated that time from p-tau217 onset explained more variability in tau PET accumulation and cognitive decline than time from amyloid PET onset for the Lilly assay but did not differ for the Alzpath assay. Overall, the timing between amyloid PET and p-tau217 onset (in a subset positive for both) ranged from −5.5–24.6 years.

These results suggest that plasma p-tau217 follows a predictable path once above thresholds thereby enabling estimation of p-tau217 + age and suggesting these assays may be useful for disease staging. Information regarding the timing of abnormal detection of amyloid PET, plasma p-tau217, and tau PET in relation to preclinical cognitive decline suggests that an optimal window for secondary prevention of Alzheimer’s disease may be within ten years of amyloid PET positivity and within five years of plasma p-tau217 positivity. Future work is needed to identify sources of observed interindividual heterogeneity in the timing of biomarker abnormalities and cognitive decline and impairment following biomarker positivity.

## Teaser

Cody, *et al*., use temporal modelling to show that Alzpath and Lilly plasma p-tau217 can provide individualized estimates of p-tau217 positive age and time, and that plasma p-tau217 positivity occurs ∼4–5 years after amyloid PET positivity and ∼3–4 years before tau PET positivity with high inter-individual variability in this timing.

## Graphical Abstract

**Graphical Abstract:**
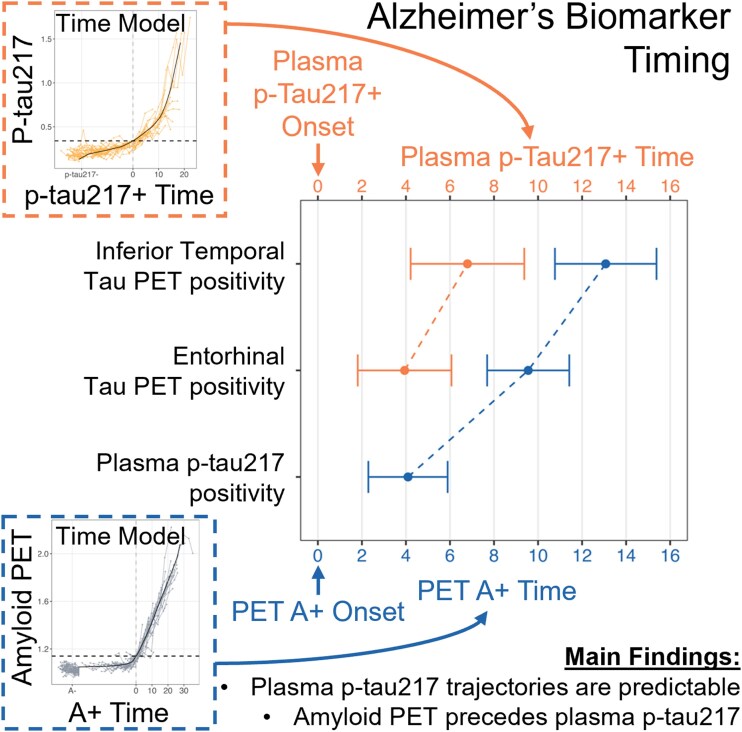

## Introduction

Neuroimaging and fluid biomarkers of Alzheimer’s disease pathology, including amyloid and tau PET and cerebrospinal fluid (CSF) and plasma assays for Aβ_40_, Aβ_42_ fragments and phospho-tau (p-tau) species (e.g. p-tau181, p-tau217, and p-tau231) have played a critical role in understanding and characterizing the natural history of disease pathology and advancing clinical trials for disease modifying therapies. Correspondingly, the Alzheimer’s Association Workgroup recently proposed a biomarker-based diagnostic framework, where abnormal Core 1 biomarkers (amyloid PET, approved CSF, and ‘accurate’ plasma biomarkers; e.g. plasma p-tau217) are sufficient to diagnose Alzheimer’s disease in the absence of symptoms, whereas abnormal Core 2 biomarkers (e.g. tau PET), presumed to change later in disease and to be more temporally proximal to symptoms, can provide prognostic information and increase confidence that Alzheimer’s disease is contributing to symptoms. Notably, these criteria have drawn criticism—particularly around diagnosing cognitively unimpaired people with abnormal Core 1 biomarkers as having Alzheimer’s disease—highlighting the need to better understand which abnormal Core 1 biomarkers can reliably predict downstream disease symptoms. A better understanding of the extent to which Core 1 biomarkers can predict if and when presumed downstream Core 2 biomarkers (i.e. tau PET) and cognitive decline will occur is needed to help clarify the appropriate use of Core 1 biomarkers in unimpaired individuals.

Recently, plasma p-tau217, has emerged as a potential scalable and reliable early indicator of abnormal amyloid and tau in Alzheimer’s disease.^1-4^ Recent studies have shown that p-tau217 in CSF and plasma distinguishes Alzheimer’s disease from non-Alzheimer’s disease dementia and distinguishes amyloid-negative (A−) unimpaired controls from amyloid-positive (A+) unimpaired as well as A + with mild cognitive impairment and dementia.^5-8^ In addition, several plasma p-tau217 assays are able to differentiate A+/− and T+/− (based on PET imaging) during the preclinical timeframe.^4,7,9-12^ While this high agreement is promising, it remains unclear when in the preclinical timeframe plasma p-tau217 is changing relative to other established biomarkers and cognitive changes. Information about relative biomarker timing and the consistency of this timing will likely be important to inform diagnostic testing for Alzheimer’s disease pathology in preclinical Alzheimer’s disease as trials and treatment progress towards secondary prevention and as anti-tau and other therapies are developed.

Our group and others have recently developed and validated temporal modelling methods that enable person-level estimates of amyloid plaque onset age ascertained from amyloid PET imaging.^13-16^ These methods and this biomarker clock framework provide a way to visualize and study the timing of key Alzheimer’s disease events, such as beta-amyloid plaque and tau tangle deposition, on an individual person level. Recent studies applying this approach to amyloid PET imaging have shown that tau tangle deposition as measured by PET imaging generally begins within ten years amyloid PET onset.^17^ Additionally, these studies show that the time from A + onset to dementia varies widely between individuals—from being nearly coincident to more than 20 years.^14,15^

By comparison, less is known about the relative timing of plasma p-tau217 and its cascade relative to changes in amyloid and tau PET and cognition in Alzheimer’s disease. While the precise temporal relationship of p-tau217 to amyloid versus tau brain tissue changes remains unresolved, the leading hypothesis suggests that amyloid pathology induces neuronal tau phosphorylation and secretion, which is reflected in biofluids, before the development of tau tangle pathology detectable via PET imaging.^18^ In symptomatic disease stages, there is also evidence of a relationship between p-tau concentrations in biofluids and tau pathology by PET^1^; however, discrepancies in early disease stages remain.^19^ With the scalability of plasma p-tau217 and its inclusion in the recent biomarker-based diagnostic framework, there is a need to understand the comparative timing and detection of elevated plasma p-tau217 relative to amyloid as well as downstream tau PET and cognitive decline.

In this work, we used novel time modelling methods in a mostly cognitively unimpaired cohort to demonstrate that plasma p-tau217, measured by Lilly Meso Scale Discovery (MSD) and Alzpath Quanterix, can be modelled to produce individual level estimates of p-tau217 + age and time, plasma p-tau217 positivity is generally an intermediate step between amyloid and tau PET positivity but this timing is highly variable between individuals, and that time from p-tau217 onset is strongly associated with brain tau pathology and cognitive decline. Specifically, we aimed to: (i) investigate temporal modelling for plasma p-tau217, (ii) characterize the timing of ^11^C-PiB amyloid PET onset and plasma p-tau217 onset relative to each other; (iii) examine associations between the timing of amyloid PET and plasma p-tau217 with tau PET accumulation, and (iv) investigate associations between the timing of amyloid PET and plasma p-tau217 onset with preclinical cognitive decline. All primary analyses were conducted using plasma p-tau217 measured with the Lilly MSD assay. To evaluate the robustness of our findings and confirm that p-tau217 timing estimates were not specific to this assay, a within-subject replication was conducted by replicating analyses with person- and time-matched plasma samples assayed using the Alzpath p-tau217 Simoa assay on the Quanterix HDX platform.

## Materials and methods

### Participants and study sample

Data were from individuals enrolled in the Wisconsin Registry for Alzheimer’s Prevention (WRAP), a longitudinal observational cohort study of adults enriched for risk of AD. WRAP participants undergo biennial neuropsychological, health, and medical history assessments.^20^ One hundred and seventy-two WRAP participants who underwent one or more cognitive assessments, were cognitively unimpaired at their baseline cognitive assessment in this analysis, and had available amyloid PET and plasma p-tau217 data were included in this study. Participants and samples for p-tau217 assays were selected such that half of the participants were A + at their last amyloid PET scan. A subsample of 148 individuals had at least one tau PET scan (Supplemental Table 1). All study procedures were approved by the University of Wisconsin-Madison Institutional Review Board and conducted in accordance with the Helsinki declaration.

### Neuroimaging methods

Detailed methods for PET radioligand synthesis and PET and MR image acquisition, processing and quantification and analysis were implemented as reported previously.^21-23^ Magnetic resonance images were obtained on a 3T GE Signa 750. PET images were acquired on either a Siemens Biograph Horizon PET/CT or a Siemens ECAT HR + tomograph.

#### Structural MRI and ROI generation

T1-weighted and T2-weighted images were tissue class segmented using spm12’s multispectral unified segmentation (https://www.fil.ion.ucl.ac.uk/spm/). Deformations obtained from the segmentation were applied to the Automatic Anatomic Labelling (AAL; for PiB) or the Harvard-Oxford (HO; for MK-6240) atlases to obtain regions of interest (ROIs) in native MR space, which were restricted to voxels with p_GM_ > 0.30. Reference region ROIs were generated by smoothing and then eroding cerebellum GM (for PiB) and inferior cerebellum GM (for MK-6240) as previously reported.^23^

#### Amyloid and tau PET imaging

Amyloid burden was assessed using a 70-min dynamic [^11^C]Pittsburgh Compound B (PiB) PET acquisition beginning with a nominal 555 MBq bolus injection. Distribution volume ratio (DVR) was calculated and a cortical average was obtained across eight bi-lateral brain ROIs (Logan graphical analysis, cerebellum grey matter reference region, *k̅*_2_ = 0.149 min^−1^, t* = 35 min).^24-26^ Tau burden was assessed 70–90 min (4 × 5-min frames) post [^18^F]-MK-6240 bolus injection. Standard uptake value ratio was calculated using the inferior cerebellum as a reference region.^22^ Regional tau burden was assessed in the entorhinal cortex, a region of early tau deposition, and the inferior temporal gyrus, a region associated with later stage tau deposition. For both PiB and MK-6240, summed PET images were used to register dynamic PET data to native space MRI.

### Biofluid methods

Blood sample acquisition, processing, and storage in WRAP have been previously described.^4^ WRAP participant plasma sample selection for analysis was based on having recently completed or being scheduled to complete an amyloid PET scan. Plasma p-tau217 concentration was measured at the Clinical Memory Research Unit, Lund University (Sweden) using an immunoassay developed by Lilly Research Laboratories on a Meso Scale Discovery (MSD) platform.^6^ Samples were assayed in duplicates according to published protocols, and the mean intra-assay coefficient of variation (CV) was 7.11%. Three quality control samples (QC, high, intermediate and low) were included in every plate. For each QC, inter-plate CV was calculated based on the mean and SD across all the plates. The mean inter-plate CV was 10.3%. One A- participant with abnormally high plasma p-tau217 concentrations was excluded from the primary analyses but was included in supplementary analyses reported in the supplement. Main findings were similar with or without this participant. All samples were analysed by staff blind to clinical and imaging data. In addition, matched plasma samples from different aliquots were also assayed using the Alzpath Simoa p-tau217 assay on a Quanterix analyser at the University of Wisconsin-Madison Alzheimer’s Disease Research Center Biomarker Lab (see Supplemental Methods for details).

### Neuropsychological testing

Cognition was assessed serially using a three-test variant of the Preclinical Alzheimer’s Cognitive Composite (zPACC-3).^27^ The WRAP zPACC-3 is derived from the Rey Auditory Verbal Learning Test total learning score (RAVLT; Trials 1–5), WMS-R Logical Memory delayed recall score, and the WAIS-R Digit Symbol test score.^28^

### Biomarker positivity and temporal modelling

PiB PET and plasma p-tau217 positivity thresholds were defined using group-based trajectory modelling (GBTM) in a two-step approach.^13,14^ First, using data from participants with longitudinal (≥2) biomarker observations (p-tau217, *n* = 162; PiB, *n* = 155), GBTM was applied separately to each biomarker to identify non-accumulating groups for plasma p-tau217 and PiB PET (e.g. the subset with no values greater than or equal to the intercept beta + 2 standard error). Second, the within-person standard deviations (SDs) of those GBTM classified as non-accumulating were used to define biomarker positivity, such that plasma p-tau217 or PiB PET positivity was defined as the GBTM non-accumulating group intercept + 95th centile of the within-person SDs of the non-accumulating group (plasma p-tau217+ > 0.34 pg/mL; A + > 1.14 DVR). Sampled iterative local approximation (SILA; https://github.com/Betthauser-Neuro-Lab/SILA-AD-Biomarker)^14^ was used to model longitudinal PiB and p-tau217 data and to estimate individual PiB and p-tau217 onset ages and durations of biomarker positivity using the positivity thresholds derived from GBTM. Briefly, SILA uses discrete sampling and Euler’s method to model biomarker accumulation over time, anchoring time zero at the respective threshold for biomarker positivity. The algorithm then estimates each participant’s age of biomarker onset by aligning their observed values at a reference observations to this modelled curve to estimate biomarker positive time and then adds this estimated time to the age at the reference observation (estimated biomarker onset age = age at observation + estimated biomarker positive time). Reference observations for individualized amyloid and p-tau217 onset age estimates were the participant’s first observation after crossing the positivity threshold for cases that were observed biomarker-positive during the study or the last observation for those who remained biomarker-negative.^14^ The first positive observation was used to avoid the need to extrapolate beyond the observed longitudinal data at the highest values of the distributions. Estimated onset ages for PiB and p-tau217 were then used to calculate the estimated duration of biomarker positivity by subtracting the participant’s age at each study visit from their estimated biomarker onset age. To ensure our findings regarding p-tau217 timing and SILA modelling were not specific to the Lilly MSD assay, we also applied SILA modelling to person- and time-matched (i.e. paired) plasma samples assayed using the Alzpath p-tau217 Simoa assay run on a Quanterix HDX analyser (Supplemental Methods). Root mean squared error is reported for SILA concentration estimation. Accuracy of SILA-estimated versus ‘observed’ p-tau217 + age are reported for both assays in subsets of observed p-tau217 negative to positive converters (see supplemental methods and results for detailed characterization of SILA model performance and estimation accuracy, Supplemental Figs 1–6).

### Statistical methods

Statistical analyses were performed in R v4.0.5. Primary analyses were conducted using plasma p-tau217 from the Lilly MSD platform and reported in the main manuscript. Secondarily, to evaluate the robustness of our findings, analyses were repeated in the same sample using plasma p-tau217 from Alzpath Quanterix and detailed in the Supplement.

Differences in amyloid and p-tau217 onset ages were assessed across *APOE* genotype and sex using independent samples *t* tests for those that were biomarker positive. To characterize the time between A + and p-tau217+, we examined the difference between A + onset and p-tau217 + onset in the subset of individuals (*n* = 61) who were plasma p-tau217 and amyloid PET-positive by their last available observations. Pearson’s correlations were used to examine the associations of A + and p-tau217 + onsets, respectively, with the time interval between A + and p-tau217 + onset.

For all tau PET analyses, we used the SILA estimated PiB and p-tau217 durations and levels at the time of the tau PET scan. In the tau PET imaging subset who were p-tau217 + and A + (*n* = 54 participants; *n* = 103 tau PET observations), we examined regional tau PET accumulation as a function of age, A + time, and p-tau217 + time at the time of the tau PET scan using mixed effects models, where each model had the same number of predictors and included random intercept as well as random age or biomarker time slope. Models were compared using corrected Akaike information criteria (AICc), marginal R^2^ and likelihood ratio tests to assess the predictor that explained the most variance in tau PET accumulation as well as whether one time operationalization resulted in better model fit than another. In the tau PET imaging subset who were A + (*n* = 79, *n* = 143 tau PET observations), we used the Wald interval (delta method) to estimate the average number of years (95% confidence interval) from amyloid onset to p-tau217 positivity as well as the average number of years from amyloid and p-tau217 onset (separately for amyloid and p-tau217) to regional tau positivity (entorhinal tau positivity >1.27 SUVR; inferior temporal gyral tau positivity > 1.42 SUVR; thresholds previously derived from WRAP data as described here^17^).

To evaluate the differential relationships of age, p-tau217 + time, and A + time with preclinical cognitive decline, we used linear mixed effects models (random intercept and random slope) in the subset of initially unimpaired p-tau217 + and A + participants (n ppts = 61; n, zPACC-3 obs = 308). Each model included one of the following time-varying predictors of interest: linear and quadratic age at zPACC-3, linear and quadratic p-tau217 + time at zPACC-3, or linear and quadratic A + time at zPACC-3. All models covaried for age at baseline zPACC-3, sex, and the number of prior exposures to the cognitive battery (i.e. practice effects). Model fits were compared using corrected Akaike information criteria (AIC); nested models were also compared with likelihood ratio tests. Statistical significance was inferred at *P* < 0.05. Post hoc comparisons of modelled zPACC-3 decline at 5 and 10 years of biomarker time were estimated using R package *ggpredict*.

## Results

### Participant characteristics

Demographic, clinical, and summary biomarker characteristics of the sample are reported in Table 1. By design, approximately half (51.2%) of the sample was A + at their most recent amyloid PET scan. At their baseline plasma assessment, participants in the study were on average 63.2 (SD = 6.3) years old, 65% were female, and 48% were *APOE-*ε4 carriers. Participants had on average 3 plasma assessments over 5.1 (SD = 1.8) years of plasma follow-up, and 3 amyloid PET scans over 7.2 (SD = 4.0) years of amyloid PET follow-up.

**Table 1:** Participant characteristics

Characteristic	Overall N = 172	Most recent amyloid PET status	
		A- N = 84	A + N = 88
Age at baseline plasma visit	63.2 (6.3)	62.0 (6.9)	64.3 (5.3)
Years of plasma follow-up	5.1 (1.8)	5.0 (1.9)	5.2 (1.8)
No. plasma visits, Median [IQR]	3 [3,4]	3 [3,4]	3 [3,4]
Age at baseline amyloid PET visit	63.1 (6.9)	60.9 (6.8)	65.2 (6.3)
Years of amyloid PET follow-up	7.2 (4.0)	7.6 (3.3)	6.8 (4.4)
No. amyloid PET scans, Median [IQR]	3 [2,4]	3 [2,4]	3 [2,4]
Age at baseline PACC-3 visit	59.7 (6.0)	58.5 (6.7)	60.7 (5.2)
Years of PACC-3 follow-up	10.6 (3.2)	10.8 (2.6)	10.5 (3.7)
No. PACC-3 assessments, Median [IQR]	5 [4,6]	5 [5,6]	5 [4,6]
Female	111 (65%)	55 (65%)	56 (64%)
*APOEe4* carriers	82 (48%)	28 (33%)	54 (61%)
Race			
American Indian or Alaska Native	3 (2%)	2 (2%)	1 (1%)
Black or African American	5 (3%)	2 (2%)	3 (3%)
White	164 (95%)	80 (95%)	84 (95%)
Education (years)	16.3 (2.1)	16.2 (2.1)	16.4 (2.0)
Clinical diagnosis at most recent visit			
Cognitively unimpaired	149 (87%)	84 (100%)	65 (74%)
MCI	17 (10%)	0 (0%)	17 (19%)
Dementia	6 (4%)	0 (0%)	6 (7%)

Values shown as mean (standard deviation) or N (%) unless otherwise noted.

Abbreviations: A−/+, amyloid PET positivity; *APOE*, apolipoprotein; PACC-3, Three-test Preclinical Alzheimer’s Cognitive Composite; PET, positron emission tomography.

### Temporal modelling of plasma p-tau217

**Figure 1:**
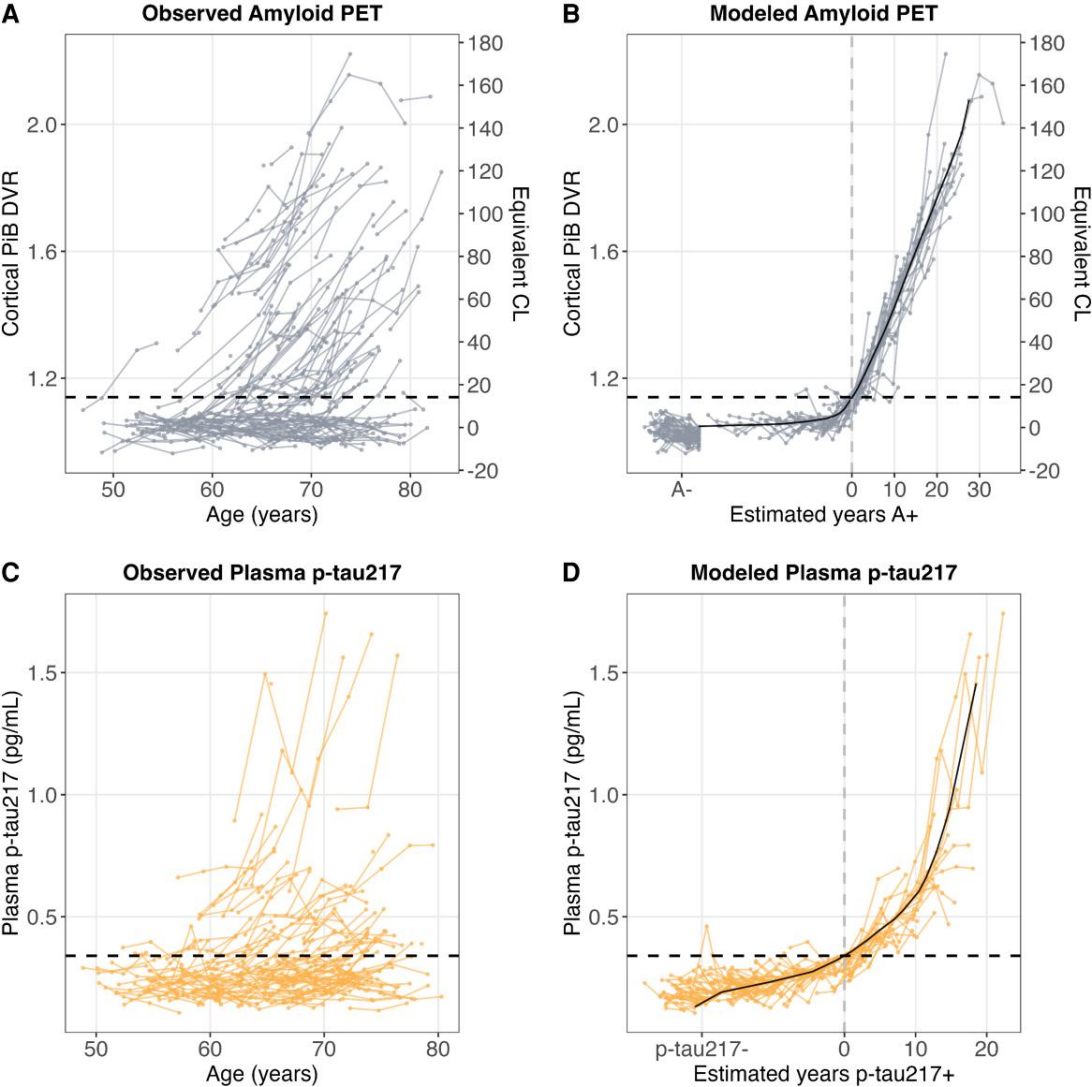
**Observed and modelled amyloid PET and plasma p-tau217 trajectories.** Lines represent longitudinal observations (connected dots) within individual participants [*n* = 172 participants; *n* = 555 PiB PET observations (**A and B**); *n* = 527 p-tau217 observations (**C and D**)]. Observed amyloid PET, shown as cortical Pittsburgh Compound B (PiB) distribution volume ratio (DVR) and equivalent centiloids (CLs; A), and plasma p-tau217 (**C**) as a function of age and the resultant amyloid (**B**) and p-tau217 (**D**) accumulation trajectories from the SILA model (solid black lines). SILA-modelled curves (**B**) and (**D**) are discrete, non-parametric curves that are the result of integrating discretely sampled rate versus biomarker value curves. Zero years for modelled amyloid PET and plasma p-tau217 timelines (grey vertical dashed lines) were defined as the point the model intersected the positivity threshold (black horizontal dashed line) for each biomarker (PiB DVR = 1.14 (14.1 Equivalent Centiloids (CL)); p-tau217 = 0.34 pg/mL). Supplemental analyses including the Lilly MSD outlier are detailed in Supplemental Fig. 7.

Biomarker positivity (+/−) thresholds defined using group-based trajectory modelling were 1.14 DVR (equivalent to 14.4 CL) for amyloid PET and 0.34 pg/mL for plasma p-tau217. Observed and SILA-modelled longitudinal biomarker trajectories and biomarker positivity thresholds are shown for cortical amyloid PET (PiB DVR) and plasma p-tau217 in Fig. 1. Across observed amyloid PET and plasma p-tau217 trajectories, both biomarkers had visually apparent groups of non-accumulators (below the biomarker-positive thresholds with slopes near zero) and accumulators (individuals crossing and/or above the biomarker-positive thresholds with positive slopes; Fig. 1A and C). In this sample, 51% (88/172) of participants showed evidence of elevated brain amyloid (A+; global PiB DVR > 1.14; Fig. 1A); 38% (66/172) were p-tau217+ (p-tau217 > 0.34 pg/mL; Fig. 1C), and 35% (61/172) were both A + and p-tau217 + at their most recent observation. For both amyloid PET and plasma p-tau217, SILA converged on an increasing curve with a relatively constant slope above their respective positivity thresholds. Time-aligned observations as a function of SILA-modelled years A + and years p-tau217+ (Fig. 1B and D) showed consistent accumulation patterns across individuals within each biomarker. Similar patterns were observed when including the p-tau217 outlier (Supplemental Fig. 7). Repeated analyses in the person- and time-matched p-tau217 plasma samples from the Quanterix HDX analyser indicated that Alzpath p-tau217 was also produced a similarly increasing curve above the positivity threshold (Supplemental Methods). Root-mean squared error for SILA estimated p-tau217 concentrations was 0.075 pg/mL for Lilly and 0.179 pg/mL for Alzpath assays. In subsets of observed p-tau217- to p-tau217 + converters, the mean difference (95% CI) between SILA-estimated p-tau217 + age and the age midpoint between the last p-tau217- and first p-tau217 + observation was −0.95 (−1.77, −0.13) years for Lilly (*n* = 27) and −0.58 (−1.87, 0.71) years for Alzpath (*n* = 23; see supplemental methods for detailed analyses). SILA-estimated p-tau217 + ages for the *n* = 66 p-tau217 + individuals were 2.4 (SD = 4.65) years later for Alzpath p-tau217 compared to Lilly MSD p-tau217 (Supplemental Methods).

### Amyloid PET positivity typically precedes plasma p-tau217 positivity

**Figure 2:**
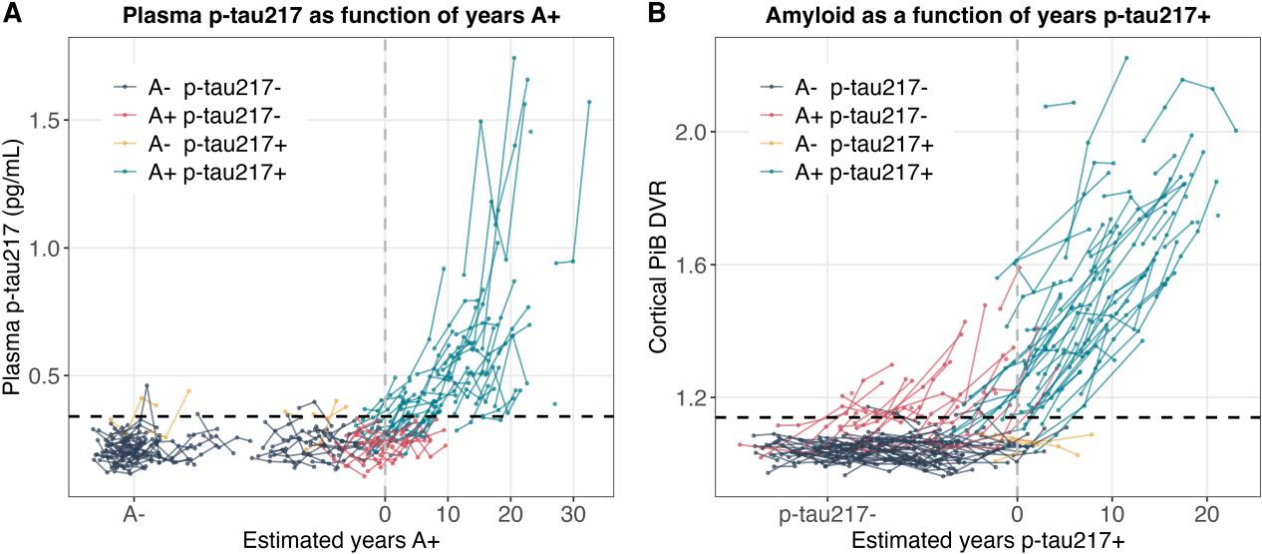
**Amyloid PET onset generally precedes plasma p-tau217 onset.** Participants were classified as biomarker positive or negative based on their last observed biomarker assessment, which resulted in four biomarker groups based on amyloid positivity (A+/−) and p-tau217 positivity (p-tau217+/−): A- p-tau217− (*n* = 79; navy), A + p-tau217- (*n* = 27; pink), A- p-tau217+ (*n* = 5; yellow), A + p-tau217+ (*n* = 61; blue). Lines represent longitudinal observations (connected dots) within individual participants [*n* = 172 participants; *n* = 527 p-tau217 observations (**A**); *n* = 555 PiB PET observations (**B**)]. Dashed black horizontal lines indicate the plasma p-tau217 positivity threshold (A; p-tau217 = 0.34 pg/mL) and cortical PiB DVR positivity threshold (B; PiB DVR = 1.14). Dashed grey vertical lines indicated the onset of A + (A) and onset of p-tau217+ (**B**). In panel (**A**), observed plasma p-tau217 as a function of estimated A + time (years) demonstrates that most individuals become p-tau217+ (i.e. crossing the horizontal black dashed positivity threshold) after amyloid onset (i.e. years A + =0; Grey vertical dashed line). In panel (**B**), observed brain amyloid as measured by cortical PiB DVR as a function of estimated years p-tau217 + demonstrates that many individuals become A + (i.e. crossing the horizontal black dashed positivity threshold) before p-tau217 onset (i.e. years p-tau217+=0; Grey vertical dashed line).

**Figure 3:**
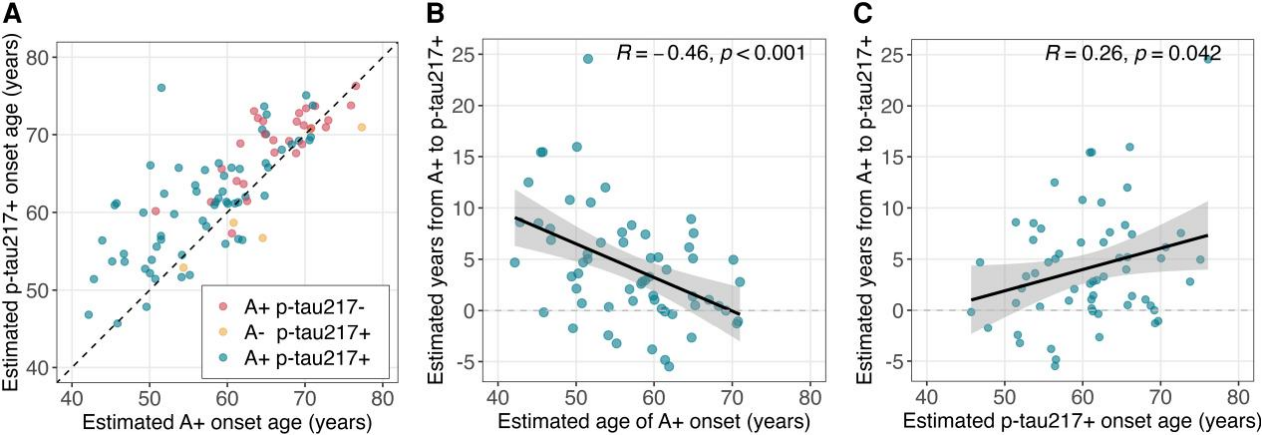
**Comparison of amyloid PET and plasma p-tau217 onset ages**. A comparison of biomarker onset ages (**A**) for individuals that became biomarker positive (A + = amyloid positive, p-tau217+ = plasma phosphor-tau217 positive) on at least one biomarker (e.g. A + p-tau217-, *n* = 27 (pink); A- p-tau217+, *n* = 5 (yellow); and A + p-tau217+, *n* = 61 total (teal). Individuals above the dashed grey diagonal line had an A + onset age that preceded their p-tau217 + onset age. For the group that was observed to be positive on both biomarkers (e.g. A + p-tau217+, teal; *n* = 61), the estimated time difference between A + onset and p-tau217 + onset is shown as a function of A + onset age (**B**) and p-tau217 + onset age (**C**) with Pearson’s R and the significance of the correlation shown at the top of each plot. In this A + p-tau217 + subset, A + onset generally preceded p-tau217 + by an average(SD) 4.3(5.6) years. A significant negative correlation was observed between time from A + onset to p-tau217 onset and age of A + indicating that a younger estimated age at A + onset was associated with longer time to p-tau217 + onset.

Plotting observed plasma p-tau217 concentrations as a function of estimated years A + (Fig. 2A, Supplemental Figs 8 and 9) suggested most individuals become p-tau217 + after amyloid onset (i.e. observed p-tau217 concentrations cross the p-tau217 threshold at A + times >0 years). Conversely, plotting observed amyloid burden (cortical PiB DVR) as a function of estimated years p-tau217 + shows several individuals becoming A + at p-tau217 + time <0 years (Fig. 2B, Supplemental Figs 8 and 9). Comparisons of individual-level estimated A + and p-tau217 + onset ages also suggested amyloid positivity generally precedes plasma p-tau217 positivity (Fig. 3A) with 76% (71/93) having estimated A + onset before estimated p-tau217 onset among the 93 participants with at least one positive biomarker. For those that were both A + and p-tau217+ (*n* = 61), the average estimated time from A + onset to p-tau217 + onset was 4.25 (SD = 5.56) years, with older A + onset age associating with a shorter time from A + onset to p-tau217 + onset (*P* < 0.001; Fig. 3B). Similar timing results were observed with plasma p-tau217 measured using Alzpath Quanterix; for example, among those who were both A + and Alzpath p-tau217+ (*n* = 54) the average estimated time from A + onset to Alzpath p-tau217 + onset was 4.61 (SD = 5.20) years, with older A + onset age associated with shorter time from A + onset to p-tau217 + onset (Supplemental Fig. 10). Neither sex nor *APOE*ε4 carriage were associated with time from A + onset to p-tau217 onset (*P* = 0.72 and 0.48, respectively). Notably, we observed substantial heterogeneity in A + onset age (range 42.2 to 76.6 years; *n* = 88 A+), p-tau217 + onset age (range 45.7 to 76.1 years; *n* = 66 p-tau217+), and the time between estimated A + and p-tau217 + onset age (range −5.5 to 24.6 years; *n* = 61 A + and p-tau217+). Similar variability in p-tau217 + onset age was observed with Alzpath p-tau217 age of onset estimates (Supplemental Fig. 10).

### Amyloid and plasma p-tau217 positivity precede tau PET positivity

**Figure 4:**
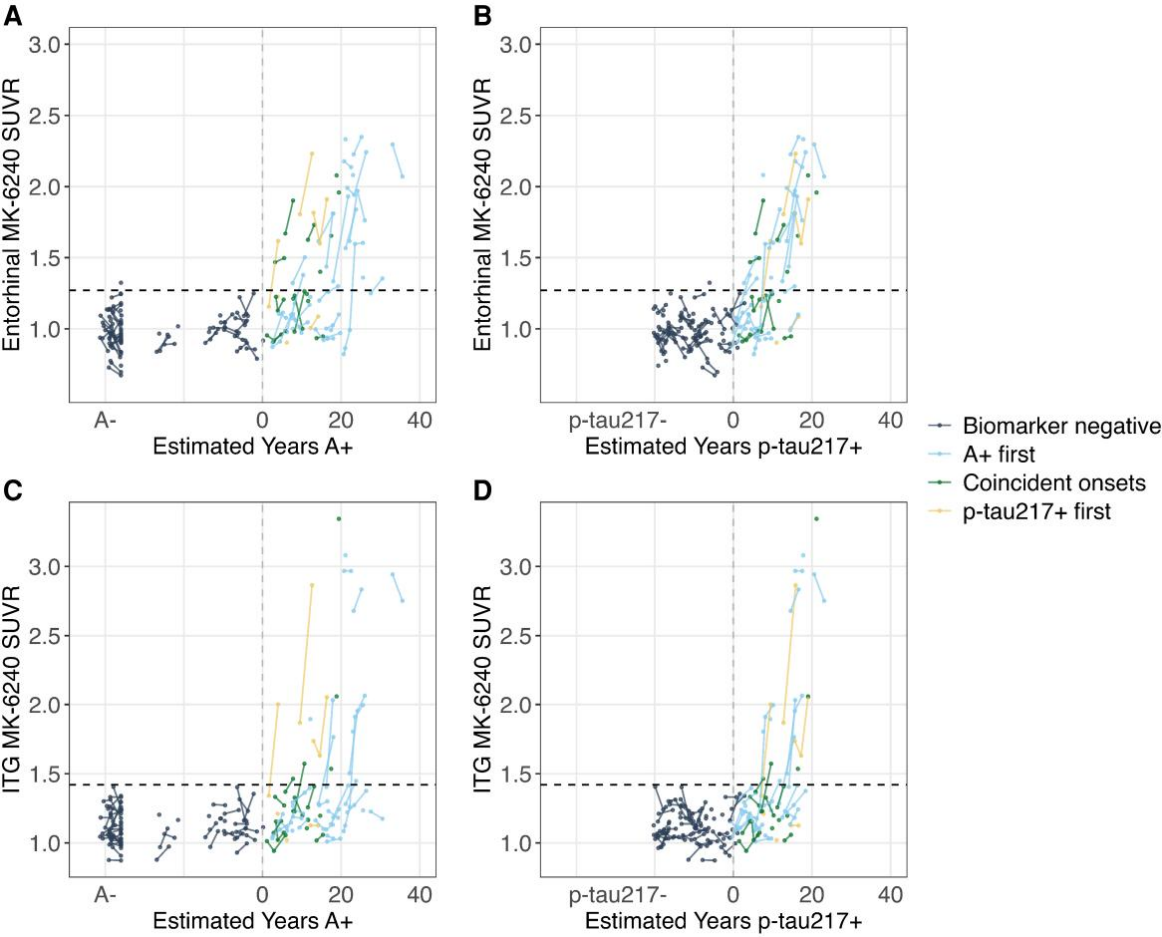
**Tau PET accumulation as a function of biomarker time.** Observed entorhinal cortex (**A** and **B**) and inferior temporal gyrus (ITG; C-D) MK-6240 tau PET standardized uptake value ratio (SUVR) plotted as a function of estimated years A + (A, C) and estimated years p-tau217+ (**B** and **D**) for participants observed to be A- p-tau217- (*n* = 67 participants) or A + p-tau217+ (*n* = 54 participants) at their last biomarker observations who had available tau PET imaging. See Supplemental Fig. 11 for participants who were censored due to having only one positive biomarker). Lines represent longitudinal tau PET observations (connected dots) within individual participants (*n* = 121 participants; *n* = 258 tau PET observations).The horizontal black dashed lines indicate regional tau PET positivity thresholds, vertical grey dashed lines indicate amyloid onset (i.e. Years A + =0) and p-tau217 + onset (i.e. Years p-tau217+ = 0), and the colours indicate relative biomarker timing: Biomarker negative (navy, *n* = 67), A + first (A + onset age > 2 years before p-tau217 + onset age; light blue, *n* = 32), Coincident onsets (A + and p-tau217 + onsets within 2 years; green, *n* = 16), and p-tau217 + first (p-tau217 + onset age > 2 years before A + onset age; yellow, *n* = 6). Repeated analyses with the Alzpath data are shown in Supplemental Figs 12 and 13.

In the subset with available ^18^F-MK-6240 tau PET imaging whose biomarker sequence could be inferred (i.e. A- p-tau217- and A + p-tau217+; *n* = 121 participants, 258 tau PET observations), we separately characterized the accumulation of brain tau in the entorhinal cortex and the inferior temporal gyrus as a function of A + time and plasma p-tau217 time. These brain regions were chosen because entorhinal cortex is often the area where tau aggregation is detected first, whereas inferior temporal gyrus is a location associated with later stage tau deposition. Biomarker timing groups were established to depict the sequence of A + first (*n* = 32), p-tau217 + first (*n* = 6), or coincident A + and p-tau217 + onset (defined as A + and p-tau217 + onsets within 2 years of each other; *n* = 16). Figure 4 displays entorhinal tau PET and inferior temporal tau PET accumulation as a function of A + time (Fig. 4A and C) and as a function of p-tau217 + time (Fig. 4B and D) and coloured by relative biomarker timing (see Supplemental Fig. 11 for participants who were censored due to having only one positive biomarker). These plots demonstrate entorhinal tau PET positivity (crossing the horizontal dashed line; Fig. 4A and B) generally occurs after both A + onset and plasma p-tau217 + onset, indicating that for individuals in the Alzheimer’s disease continuum, amyloid and plasma p-tau217 are becoming abnormal before the earliest detectable changes of tau aggregation. Repeated analyses with the Alzpath data indicated similar biomarker timing, such that both amyloid and plasma Alzpath p-tau217 became abnormal before the earliest changes in tau PET burden (Supplemental Figs 12 and 13).

Among A + ptau217 + individuals (*n* = 54; n, tau observations = 103), we compared mixed effects models of age, A + time and p-tau217 + time predicting regional tau PET accumulation. In separate models, age, A + time, and p-tau217 + time at tau PET significantly predicted regional tau PET accumulation (all *P* < 0.001), and model comparisons indicated that plasma p-tau217+ time explained more variance compared to A + time and age in both entorhinal (p-tau217 + time R^2^ = 0.48; A + time R^2^ = 0.41, age R^2^ = 0.13) and inferior temporal (p-tau217 + time R^2^ = 0.41; A + time R^2^ = 0.32, age R^2^ = 0.08) gyrus tau PET trajectories (Supplemental Table 2). Parallel analyses using Alzpath p-tau217 + time showed that Alzpath p-tau217 + time and A + time explained more variance in tau PET accumulation compared to age, but did not differ significantly from one another (Likelihood ratio tests, *P* > 0.05; Supplemental Table 3).

**Figure 5:**
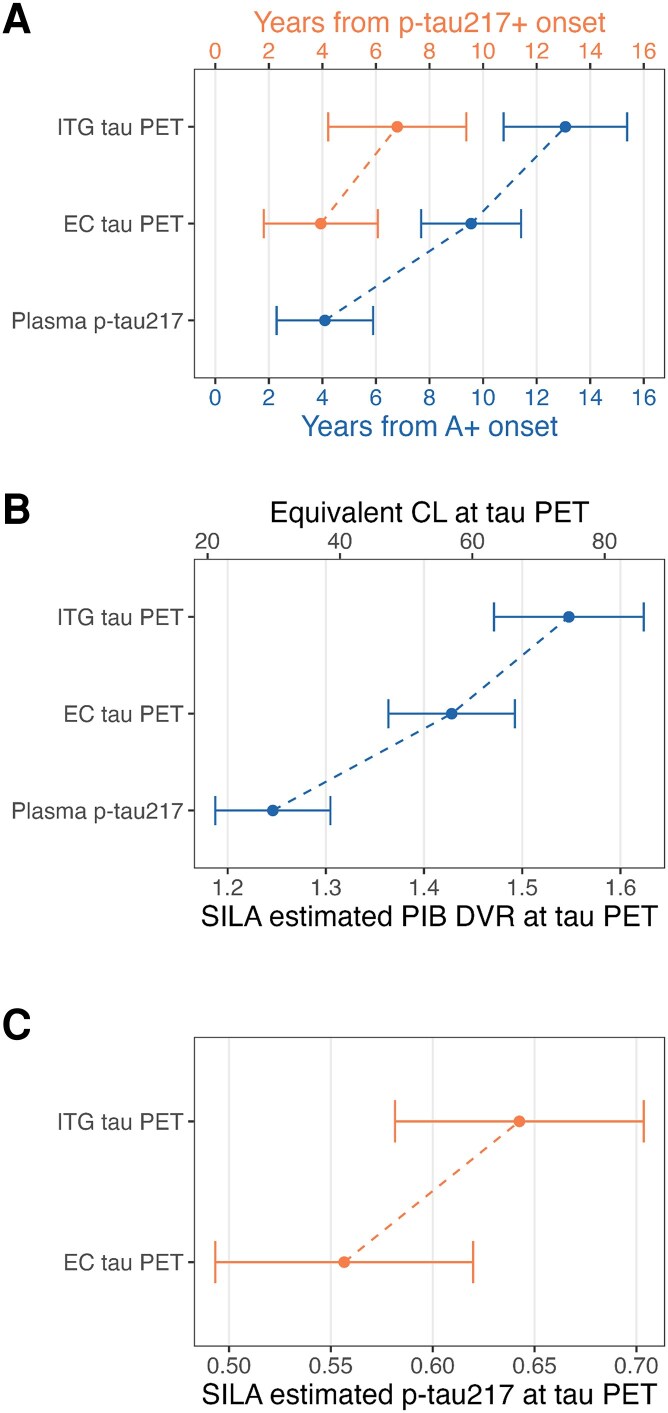
**Relative timing of biomarker positivity as a function of amyloid and p-tau217 time.** Estimated time to plasma p-tau217 and regional tau positivity from amyloid PET (A+) onset among A + individuals (n, participants = 79; n, tau PET observations = 143). Linear regressions of A + time predicting longitudinal plasma p-tau217 at the time of the tau PET scan, entorhinal (EC) and inferior temporal gyral (ITG) tau PET as well as linear regression of plasma p-tau217 + time predicting EC and ITG tau PET were conducted. We then used inverse regression (Wald interval, delta method) to predict time from A + to plasma p-tau217, EC tau PET, and ITG tau PET positivity as well as time from plasma p-tau217 + to EC and ITG tau PET positivity. Points indicate the estimated mean time with whiskers indicating the 95% confidence interval. (**A**) Time estimates of biomarker positivity along the amyloid timeline in blue and time estimates of tau PET positivity along the p-tau217 timeline shown in orange, with error bars representing the standard error for each time estimate. On average, the magnitude of tau biomarker changes along the amyloid timeline followed the expected pattern of tau accumulation, such that detectable changes in plasma p-tau217 occurred before detectable changes in EC tau PET accumulation which preceded detectable tau spread outside of the medial temporal lobe (ITG tau PET accumulation). For additional context, we also ran models using amyloid PET DVR/centiloids (CL) and Lilly Meso Scale Discovery (MSD) p-tau217 concentration instead of time estimates, which show the estimated amyloid DVR and p-tau217 concentrations corresponding to plasma and tau PET positivity (**B** and **C**, respectively). Repeated analyses with the Alzpath data are shown in Supplemental Fig. 14.

Estimates of downstream biomarker positivity along the amyloid and plasma p-tau217 timelines are summarized in Fig. 5 and Supplemental Fig. 14. Among A + individuals, on average plasma p-tau217 + occurred 4.1 (95% CI, 2.3–5.9) years after A + onset with entorhinal tau positivity occurring 9.7 (95% CI, 7.7–11.4) years after A + onset and inferior temporal gyrus tau positivity occurring 13.1 (95% CI, 10.8–15.4) years after A + onset. Correspondingly, when we used SILA estimates of amyloid level rather than A + time, we observed that on average, plasma p-tau217 + occurred at approximately 30 CL (95% CI, 21–39 CL), entorhinal tau PET positivity occurred at 57 CL (95%CI, 47–66 CL) and inferior temporal gyrus T + occurred at 75 CL (95%CI, 63–86 CL). In contrast, along the p-tau217 timeline, entorhinal tau PET positivity occurred on average 3.9 (95% CI, 1.8–6.1) years after p-tau217 + onset and inferior temporal gyrus tau positivity occurred 6.8 (95% CI, 4.2–9.4) years after p-tau217 + onset among A + individuals. Timing of relative biomarker positivity were nearly identical between estimates of Alzpath plasma p-tau217 time and estimates of Lilly plasma p-tau217 time (Supplemental Fig. 14).

### Amyloid time and plasma p-tau217 + time predict prospective cognitive decline

**Figure 6:**
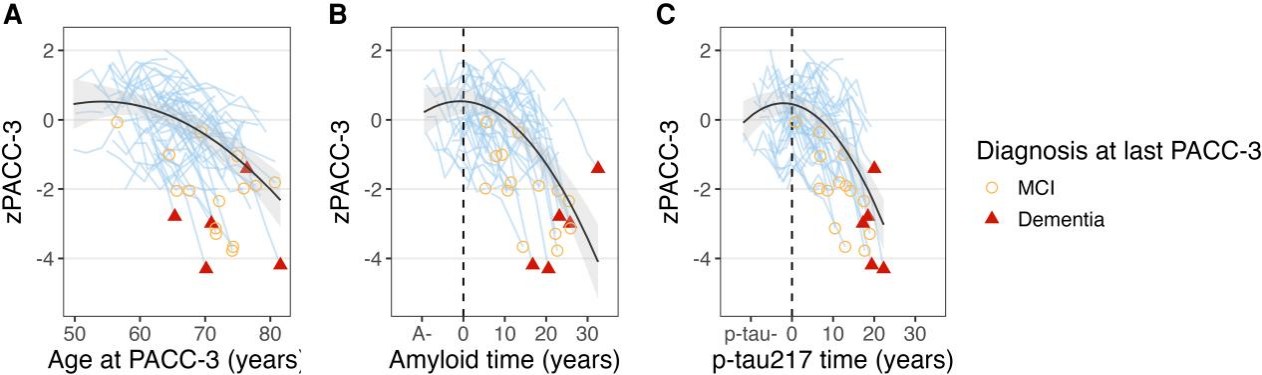
**Preclinical Alzheimer’s cognitive composite scores by age, A + time, and p-tau217 + time.** Observed and modelled preclinical Alzheimer’s cognitive composite scores (zPACC-3) by age (**A**), A + time (**B**), and p-tau217 + time (**C**) for 61 A + p-tau217 + individuals with a total of 308 zPACC-3 observations over an average 10.8 (3.9) years of zPACC-3 follow-up. Light blue lines represent observed longitudinal zPACC-3 trajectories for individual participants. All participants were cognitively unimpaired at baseline zPACC-3 and their diagnosis at their most recent zPACC-3 is indicated by shape (no shape = cognitively unimpaired; open yellow circle = mild cognitive impairment (MCI); filled red triangle = dementia). Dashed vertical lines indicate biomarker onset for amyloid (**B**) and p-tau217 (**C**), respectively. For each plot, zPACC-3 was modelled using mixed effects models including linear and quadratic time, baseline zPACC-3 age, gender, practice, random intercept and random time slope where time was mean centred and operationalized as age in panel (**A)**, A + time in panel (**B)**, and p-tau217 + time in panel (**C)**. Modelled zPACC-3 trajectories are shown as the black bold line and shaded areas indicate 95% CIs. Model comparisons are detailed in Table 2. Repeated analyses with the Alzpath data are detailed in Supplemental Fig. 15 and Supplemental Table 4.

Figure 6 shows observed and modelled cognitive (PACC-3) trajectories as a function of age, A + time, and p-tau217+ time at PACC-3 for 61 A + p-tau217 + individuals with a total of 308 PACC-3 observations over an average (SD) 10.8 (3.9) years of PACC-3 follow-up. Mixed effects models characterizing prospective preclinical cognitive decline as a function of age, amyloid time, and p-tau217 + time indicated that amyloid and p-tau217 + time at PACC-3 explained considerably more variance in longitudinal cognitive decline compared to age at PACC-3 (Table 2). Model fit comparisons indicated p-tau217 + time (AICc = 684.0) was a better predictor of preclinical PACC-3 decline compared to A + time (ΔAICc = 21.0) and age (ΔAICc = 10.1). On average (se), as a function of p-tau217 time, the rate of cognitive decline 5 years after p-tau217 onset was −0.20 (0.02) and increased to −0.26 (0.03) 10 years after p-tau217 onset. In contrast, as a function of amyloid time, the average rate of cognitive decline was −0.13 (0.02) after 5 years of A + and −0.17 (0.02) after 10 years of A + . In repeated analyses with Alzpath p-tau217 time, we observed similar results indicating p-tau217 + time explained the most variance in PACC-3 decline and was the model of best fit for predicting PACC-3 decline (Supplemental Table 4 and Supplemental Fig. 15).

**Table 2:** PACC-3 decline in a + ptau217 + individuals as a function of age, amyloid time, and p-tau217 time

Comparison of models fit in N = 61 A + p-tau217 + individuals with *n* = 308 PACC-3 observations	ΔAICc	Marginal R^2^
zPACC3 ∼ Age at PACC3^2^ + covariates + (age|participant)	10.1	0.27
zPACC3 ∼ A + time^2^ + covariates + (A + time|participant)	21.1	0.35
zPACC3 ∼ p-tau217 + time^2^ + covariates + (p-tau217 + time | participant)	0	0.35

Each model included 308 zPACC-3 observations for 61 A + ptau217 + individuals. Models included one of the following time-varying predictors of interest: linear and quadratic age at PACC-3, linear and quadratic ptau217 + at PACC-3, or linear and quadratic A + time at PACC-3. All models covaried for age at baseline PACC-3, sex, and the number of prior exposures to the cognitive battery (i.e. practice effects). All models included lower order terms. Age, A + time, and p-tau217 + time were each mean centred and significant (*P* < 0.001) quadratic and linear predictors in each model. Covariates included fixed effects of age at first zPACC-3, sex, and number of zPACC-3 exposures. All models included lower-order terms. Model were compared using corrected Akaike information criteria (AICc) and marginal R^2^.

## Discussion

The primary goal of the study was to determine if temporal modelling and age of biomarker onset could be ascertained from longitudinal plasma p-tau217 data, and secondarily, to characterize the sequence and timing of p-tau217 relative to other Alzheimer’s disease biomarkers and prospective cognitive decline. Our results provide strong evidence that SILA can be used to model longitudinal p-tau217 concentrations and estimate individualized p-tau217 + onset ages. Using individualized amyloid PET and p-tau217 onset ages, we observed that PiB PET A + onset generally preceded plasma p-tau217 positivity by ∼4 years on average, and the time from A + onset to p-tau217 + onset was shorter with older age. Using time from A+, we observed the expected pattern of biomarker accumulation, such that detectable changes in plasma p-tau217 occurred before detectable changes in entorhinal tau PET accumulation which preceded detectable tau spread outside the medial temporal lobe. Further, we observed the time from p-tau217 + onset to tau PET T + onset was shorter in the medial temporal lobe compared to the inferior temporal gyrus as is expected based on Braak NFT staging^29^ and concordant patterns observed in tau PET imaging studies.^30-32^ Additionally, in the subset that was A + and p-tau217+, we found that time from p-tau217 onset was a better predictor of prospective PACC-3 decline compared to time from A + onset or age at PACC-3. Notably, all of these findings were consistent across Lilly MSD and Alzpath Quanterix plasma platforms. Combined, these results provide critical information about the timing and progression of Alzheimer’s disease pathophysiological changes as reflected by imaging and biofluid-based biomarkers during the preclinical phase of disease that likely have significant consequences for disease diagnosis and effective treatment.

Several Alzheimer’s disease biomarker studies over the past two decades have suggested that effective treatment and secondary prevention of Alzheimer’s disease proteinopathies requires early intervention and detection of pathologic changes occurring before clinical symptoms are observed.^33,34^ This hypothesis is further supported by recent donanemab anti-amyloid trial results that showed a more pronounced treatment effect in patients with lower pre-treatment levels of tau pathology assessed by tau PET imaging.^35^ In addition, previous observational studies have shown that tau spread outside of the medial temporal lobe on PET imaging is closely aligned with the development of clinical symptoms.^31,32,36^ Our results suggest that plasma p-tau217 positivity is likely an intermediate step between aggregated amyloid and tau pathology, and therefore is likely useful for early diagnosis of preclinical Alzheimer’s disease and enrolment for secondary prevention before widespread and extensive tau pathology has accumulated. Across both Lilly and Alzpath platforms, p-tau217 + time was a strong predictor of tau PET accumulation; however, only Lilly p-tau217 + time explained significantly more variance compared to A + time. Importantly, our data suggest that among A + individuals, the time from p-tau217 + to inferior temporal T + is consistent across platforms with relatively narrow confidence intervals: Lilly p-tau217 + to inferior temporal T+=6.8 years (95% CI = 4.2 to 9.4 years) and Alzpath p-tau217 + to inferior temporal T + = 6.0 years (95% CI = 3.6–8.5) years. Combining these timing results with recent clinical trial results and what is known about tau spread and clinical symptoms suggests that this time window between p-tau217 + and inferior temporal tau positivity is likely highly important for delaying or potentially halting tau progression and thereby downstream cognitive decline and clinical impairment. Additionally, our results showing cognitive test scores as a function of p-tau217-positive time also suggest that even more subtle preclinical cognitive changes are occurring a decade or more after p-tau217 onset, again suggesting this may be a critical window for intervention. However, as a small number of participants became p-tau217 positive before amyloid PET, confirmation of pathologic amyloid by PET imaging or CSF may be required before administration of anti-amyloid therapies, particularly for those with concentrations near the positivity threshold. When considering stratification of clinical trial participants for secondary prevention based on biomarker values, our data suggests amyloid PET CL of 30 (∼4 years after PET A+) or lower might be appropriate as this was the level at which p-tau217-positivity occurred on average, which may be indicative of amyloid-related tau hyperphosphorylation. We also observed entorhinal tau positivity at 57 CL (∼9 years after A+) and inferior temporal tau positivity at 75 CL (∼12 years after A+). Combined, these data suggest that the optimal time window for anti-amyloid intervention is likely within the first decade of PET-measured A+, and within the first five years of plasma p-tau217 positivity, before measurable tau deposition occurs and before measurable cognitive deficits occur.

Our data are also consistent with the proposed mechanisms and sequence of tau aggregation in AD. For example, it has long been hypothesized and suggested from *in vitro* and animal studies that tau hyperphosphorylation results in dissociation of tau protein form the microtubule surface, accumulation of soluble hyperphosphorylated tau, and eventual aggregation of insoluble tau filaments that go on to form neurofibrillary tangles in AD.^18^ In addition, recent biomarker studies also suggest that tau hyperphosphorylation and aggregation is mediated by beta-amyloid pathology.^37,38^ Our results are highly consistent in that we observed A + PET (a measure of insoluble beta-amyloid plaque pathology) prior to increases in p-tau217 levels, followed subsequently by aggregation of tau pathology on tau PET (a measure of insoluble tau aggregates). While this is encouraging, it is worth noting that each biomarker has a different sensitivity to detect tissue changes for their respective biological targets, and therefore different detection limits may somewhat confound this interpretation. For example, imaging-autopsy studies for tau PET tracers suggest that tangle pathology is present and likely spread to Braak V or VI neocortical regions by the time medial temporal tau pathology can be detected with current tau PET radioligands.^39,40^ These types of studies in patients with extensive antemortem biomarker characterization will be crucial to inform the detectability of current biomarkers to underlying pathology and inference about pathophysiologic changes. Further, future work including mediation analysis is needed to probe potential bidirectional or sequential influences between amyloid PET, plasma p-tau217, and tau PET pathology.

Regarding p-tau217, current literature suggests that it is possible that other assays, such as those using mass spectrometry, or assays for different p-tau isoforms may be more or less sensitive to detect early pathophysiologic changes in tau phosphorylation compared to the Lilly MSD and the Alzpath assay used here.^41^ Although we did not have mass spectrometry data, we did observe that timing results were similar on average between Lilly MSD versus Alzpath p-tau217 assays, with Alzpath p-tau217 +being 2.4 years after MSD p-tau217 + albeit with notable person-level variability. It remains possible that differences in assay sensitivity and detection thresholds could contribute to the observed discrepancy in ptau217 + onset estimates between the Alzpath and Lilly MSD assays. Additionally, the Alzpath and Lilly MSD assays were used across different subsets of participants, which may introduce additional variability in measurement that complicates direct comparisons. Further, plasma biomarkers have been shown to be susceptible to confounding by comorbid conditions such as renal or hepatic dysfunction, systemic inflammation, and vascular pathology, all of which can influence protein clearance and concentration.^42^ These factors may contribute to heterogeneity in p-tau217 levels and thus heterogeneity in temporal estimates of p-tau217 + . Further work is needed to investigate whether other assays can be similarly modelled and if they provide earlier detection of Alzheimer’s disease pathophysiologic change.

Our results on average recapitulate the proposed sequence of biomarker, pathologic processes, and cognitive changes,^43^ but similar to our and other recent works,^13-15^ we observed considerable heterogeneity in the timing of these events at the individual level, especially for the time from A + PET onset to p-tau217 + and T + PET onset. The use of individualized biomarker onset ages enables more clear observation and study of the sources of this variability in disease timing. For example, several biomarker studies have shown differential risk and associations between amyloid burden measured from PET and cognitive decline and/or impairment.^44-46^ Our results suggest that these mixed findings are likely at least partly explained by the difference in when people develop amyloid pathology during their lifetime and when pathophysiologic tau hyperphosphorylation and aggregation occurs relative to amyloid onset. In both cases, age remains a significant risk factor for pathology onset and more rapid progression from A + onset to tau onset and subsequent impairment. With currently available biomarkers, it remains unclear if this is an age-related susceptibility to disease pathology, the presence of additional unmeasured/unknown brain pathology that is also age-associated, or a combination of both. The development of additional *in vivo* biomarkers for other brain pathologies such as synucleinopathy, TDP-43, and others that often co-occur with Alzheimer’s disease pathology^47^ is greatly needed to parse the effects of other neurodegenerative processes. These results regarding heterogeneity in person-level biomarker timing also support the increasing importance of biomarker panels in clinical trial selection and for personalized medicine as new biomarkers and therapies are developed.

The ability of temporal models to accurately describe longitudinal biomarker trajectories and provide individualized estimation of positivity onset age provides insights about the biological processes being measured and inferences that can be obtained from these biomarkers. Several studies have shown that longitudinal amyloid PET can be accurately modelled and can provide accurate individualized onset age estimation,^13-16^ which is largely attributed to the idea that PET binding estimates are reflective of the integral accumulation of insoluble beta-amyloid plaques over time. Interestingly, this work suggests that longitudinal p-tau217 can also be accurately modelled, but unlike PET proteinopathy tracers that measure insoluble protein aggregates directly in the tissue, p-tau217 assays measure an N-terminal soluble form of tau that may serve as an indirect biofluid-based indicator (a reporter) of C-terminal tau aggregation into insoluble tangles in the tissue (the higher the p-tau217 concentration, the more intraneuronal C-terminal tau that can be the substrate of tau aggregation). In both cases, a first-order relationship is maintained, which is useful for considering biomarkers as stage versus state markers. In addition, both processes appear to be monotone in that once abnormal levels are reached, they will continue to become more abnormal over time depicting chronic pathologic processes. The fact that p-tau217 appears to be useful for disease staging, especially extending several years into the preclinical timeframe, will undoubtedly be important for not just screening, but also staging preclinical AD. It is worth noting, that in the recently revised Alzheimer’s disease diagnosis and staging framework, plasma p-tau217 is not used for staging beyond evidence of Alzheimer’s disease pathology.^48^ A notable difference between PET and various biofluid assays is test-retest variability that also needs to be considered when assessing disease stage/progression. For example, PET reference tissue methods and binding estimates typically provide test-retest variability on the order of 5–10%,^24^ whereas test-retest variability for the Lilly p-tau217 assay has been reported at 20%.^49^ In addition, our results also suggest that since p-tau217 changes are being detected after amyloid PET, they provide a more truncated timeline and staging during preclinical AD.

This study has several strengths and weaknesses that are worth considering. A strength of the study is the rich amount and longitudinal duration of follow-up for the research participants in this study, as well as longitudinal follow-up during a key phase of preclinical disease beginning in middle- to late-middle age wherein pathology is accumulating in people that are likely at higher lifetime dementia risk as a result. A caveat for interpreting these results is the study population is largely healthy, 50% A+, mostly unimpaired, highly educated, non-Hispanic white, and enriched for Alzheimer’s disease risk. As such, the extent to which these findings will generalize to other populations is unknown. Further work is needed and ongoing to diversify study populations to ascertain whether these biomarkers, models, and risk factors will generalize more broadly to populations observed in the clinic. Additionally, studying the temporal sequence of disease requires that people exhibit abnormal levels of biomarkers during the time they are observed in clinical research studies. Some comparisons require investigating these aspects in subsets of the data that may be inherently biased. While our thresholds for detecting abnormal biomarker levels were data driven, the use of the 95th percentile threshold may underestimate clinically meaningful early or sub-threshold biomarker elevations. The temporal sequencing of amyloid and plasma p-tau217 in this study may not translate to other amyloid or tau PET tracers or other plasma p-tau217 assays. Lastly, all current tau PET tracers are confounded to some extent by off-target signal and this differs between tau tracers. As a result, these results may not generalize to other tau PET tracers.

In conclusion, longitudinal p-tau217 using the Lilly MSD and Alzpath Quanterix platforms can be accurately modelled to estimate individual p-tau217 onset ages. Aligning various biomarker and cognitive observations along biomarker timelines reveals expected patterns on average but a high degree of heterogeneity in the timing of key disease events that affect the rate of disease progression at the individual level. Future studies investigating the sources of these person-level differences will be key to defining personalized treatment strategies and intervention windows.

## Supplementary Material

fcaf449_Supplementary_Data

## Data Availability

Data for the WRAP study can be requested by qualified researchers at the following site: https://wrap.wisc.edu/data-requests-2/. SILA source code and documentation is freely available at: https://github.com/Betthauser-Neuro-Lab/SILA-AD-Biomarker.
