# Supplementary material for: Characterizing the onset and progression of Alzheimer’s pathologies using amyloid and tau PET imaging and plasma p-tau217
Source: Brain Commun. 2025 Nov 18;7(6):fcaf449. doi: 10.1093/braincomms/fcaf449 (PMC12661573; doi:10.1093/braincomms/fcaf449)
Supplement: fcaf449_Supplementary_Data [file fcaf449_supplementary_data.docx]

**Table of contents**

**Supplemental Methods**

**SILA characterization and replication using paired Quanterix Alzpath p-tau217**

- Quanterix Alzpath p-tau217 quantification
- Alzpath p-tau217 positivity threshold
- SILA modeling and time estimation
- SILA model performance characterization
- Accuracy of estimated p-tau217+ age
- Comparison of Lilly and ALZpath p-tau217+ age in the replication dataset
- Results
  - Comparisons of observed p-tau217 across platforms
    - Supplemental Figure 1
  - SILA p-tau217 trajectories and model performance
    - Supplemental Figure 2
    - Supplemental Figure 3
  - SILA estimated p-tau217+ ages in subsets of Lilly and ALZpath converters
    - Supplemental Figure 4
  - Comparisons of Lilly and ALZpath estimated p-tau217+ ages
    - Supplemental Figure 5
    - Supplemental Figure 6

**Supplemental Tables**

**Supplemental Table 1.** Tau PET subsample characteristics

**Supplemental Table 2.** Tau PET accumulation in A+ ptau217+ individuals as a function of age, amyloid time, and Lilly p-tau217 time

**Supplemental Table 3.** Tau PET accumulation in A+ Alzpath Quanterix ptau217+ individuals as a function of age, amyloid time, and Alzpath Quanterix p-tau217 time

**Supplemental Table 4.** PACC-3 decline in A+ptau217+ individuals as a function of age, amyloid time, and Alzpath p-tau217 time

**Supplemental Figures**

**Supplemental Figure 7.** Temporal modeling of amyloid PET and Lilly p-tau217 in 173 individuals from WRAP, including Lilly p-tau217 outlier

**Supplemental Figure 8.** Amyloid PET onset generally precedes Lilly plasma p-tau217 onset (including Lilly p-tau217 outlier)

**Supplemental Figure 9.** Amyloid PET onset generally precedes Alzpath plasma p-tau217 onset

**Supplemental Figure 10.** Comparison of amyloid PET and Alzpath plasma p-tau217 onset ages

**Supplemental Figure 11.** Observed Tau PET SUVR as a function of biomarker time with censored observations (Lilly p-tau)

**Supplemental Figure 12.** Observed Tau PET SUVR as a function of biomarker time (Alzpath p-tau)

**Supplemental Figure 13.** Observed Tau PET SUVR as a function of biomarker time with censored observations (Alzpath p-tau)

**Supplemental Figure 14.** Relative timing of biomarker positivity as a function of amyloid and Alzpath p-tau217 time.

**Supplemental Figure 15.** Preclinical Alzheimer’s cognitive composite scores by age, A+ time, and Alzpath p-tau217+ time.

**Abbreviations:** A+ = amyloid positive, CV = coefficient of variability, DVR = distribution volume ratio, MSD = meso scale discovery, PET = positron emission tomography, PiB = Pittsburgh compound B, p-tau217+ = plasma phospho-tau217 positive, SILA = sampled iterative local approximation, SUVR = standard uptake value ratio,

**Supplemental Methods:**

**SILA characterization and replication using paired Quanterix ALZpath p-tau217**

To ensure our findings regarding p-tau217 timing and SILA modeling were not specific to the Lilly MSD assay, we also applied SILA modeling to person- and time-matched (i.e., paired) plasma samples assayed using the Alzpath p-tau217 Simoa assay run on a Quanterix HDX analyzer (N, matched subjects = 172; N, plasma observations = 520). All Lilly observations had paired Alzpath values except for one observation, which was the last of three observations for a participant that was p-tau217+ at all plasma observations for both p-tau217 assays.

Samples and Quanterix Alzpath p-tau217 quantification: Matched EDTA plasma samples from different aliquots used for Lilly were analyzed using the ALZpath Simoa p-tau217 assay (Quanterix Part number 104570, Lot numbers 999008 and 999024) at the University of Wisconsin ADRC Biomarker Lab. Analysis was performed according to manufacturer instructions. Briefly, samples were thawed at room temperature then centrifuged for 10 minutes at 10,000 x g. The supernatant was then transferred to a 96-well plate for analysis. Each sample was measured in duplicate, and the average %CV for replicate measures was 3.8%. Manufacturer controls were analyzed in duplicate with every batch to assess batch-to-batch reproducibility. The within-plate %CV ranged from 0.1% to 18% and the between-plate %CV was 12%. A crosswalk was performed to bridge analyses across lots based on a larger sample of participants (5082 samples from N=1943 participants; Pearson r = 0.97, p <.001).

Alzpath p-tau217 positivity threshold: Because GBTM did not identify an intercept-only trajectory group for the Alzpath p-tau217 dataset, a slightly different approach was used to define the positivity threshold. For all participants in the GBTM-defined Lilly p-tau217 non-accumulating group, the within person mean and standard deviations were calculated for the Alzpath p-tau217 data. The positivity threshold for Alzpath p-tau217 was set as the mean of the within-person means in this subset plus the 95^th^ percentile of the within-person standard deviations. This yielded an Alzpath p-tau217 positivity threshold of 0.50 pg/mL. This process was repeated for the Lilly p-tau217 data to ensure this small difference in threshold definition process produced a similar threshold to that used in the primary analyses. Applying this same process to Lilly p-tau217 yielded a positivity threshold of 0.34 pg/mL, which was the same as the primary analyses (actual difference between Lilly p-tau217 thresholds was 0.0014 pg/mL).

SILA modeling and time estimation: We applied SILA to all longitudinal observations from the Alzpath p-tau217 dataset to produce a p-tau217 vs. time curve with the initial condition that time = 0 at the Alzpath p-tau217 threshold of 0.50 pg/mL. For SILA model performance characterization, we used each person’s last observation to estimate p-tau217+ time and age for both Lilly MSD and Quanterix Alzpath assays separately. We used the last observation rather than the first positive observation (as in the main analyses) to maximize the time between observations, which we felt would allow us to better characterize model accuracy over a longer time spans compared to using observations that were closer together in time. For each person, the estimated p-tau217 positive time at the reference observation was subtracted from the age at that observation to estimate the time each person crossed the p-tau217+ threshold. This process yielded two sets of p-tau217+ estimates; one from the primary analyses wherein SILA was trained on Lilly p-tau217, and a second set wherein SILA was trained on Alzpath p-tau217 data.

SILA model performance characterization

Observed Lilly and ALZpath p-tau217 concentrations from paired observations were first plotted as a function of age and as a function of each other to understand associations and discrepancies in observed values between the assays. SILA-modeled p-tau217 concentrations vs. estimated p-tau217+ time was plotted separately for each assay and visually inspected for reasonable model fits to the data. SILA model performance was characterized in two ways with analyses being conducted the same but separately for Lilly MSD and ALZpath p-tau217 assays. We used the SILA models to estimate plasma concentration at a first observation based on the observed plasma concentration at each person’s last observation. Residuals of the estimated concentrations were then plotted as a function of time from reference observation and observed concentration, and root mean squared error was calculated for both Lilly and Alzpath datasets.

Accuracy of estimated p-tau217+ age

The accuracy of the SILA-estimated p-Tau217+ onset ages for Lilly and Alzpath assays was assessed by comparing these SILA estimates to “observed” positivity ages in subsets of participants that converted from p-tau217 negative to p-tau217 positive. Converters were defined separately for each assay as participants that were initially observed to be below threshold at their first observation but became positive by their last observation. For each converter and assay, the “observed” age of positivity was calculated as the age midpoint between the age the person was last observed to be p-tau217 negative and the first observation they were observed to be p-tau217 positive. T-tests were used to determine if the difference between SILA-estimated and observed p-tau217 positive ages were non-zero, with the mean and 95% confidence interval provided for this difference for each assay separately.

Comparison of Lilly MSD and ALZpath p-tau217+ age in the replication dataset:

To be consistent with the primary analyses, p-tau217+ age and times were estimated using each person’s first positive observation (or last negative observation for those that remained p-tau217-) as a reference. Paired estimates of p-tau217+ age derived separately from Lilly and Alzpath data were compared using Bland-Altman analysis. These estimates were used for the full analysis replication later in the supplement.

**Results:**

Comparisons of observed p-tau217 data across platforms

For both Lilly MSD and Alzpath plasma p-tau217 assays, participants below the positivity thresholds had within-person slopes near zero with Alzpath p-tau217 having slight positive slopes at older ages in this group. Both assays observed generally positive within-person slopes for those above the p-tau217+ thresholds (**Supplemental Figure 1**). Scatter plots comparing Lilly and Alzpath data suggested a potentially nonlinear association between p-tau217 concentrations although there was sparse data for higher values that appeared to drive this relationship. In addition, a small number of discordant cases were observed wherein one assay was high while the other was lower compared to the general trend in paired comparisons. ALZpath p-tau217 concentrations were generally higher than Lilly p-tau217 concentrations.

SILA p-tau217 trajectories and model performance

Similar to the main findings with the Lilly assay, Alzpath p-tau217 data and SILA trajectory modeling produced a curve with overall increasing trajectory and relatively constant slope above the positivity threshold (**Supplemental Figure 2**). RMSE for concentration estimation was 0.075 pg/mL for Lilly and 0.179 pg/mL for ALZpath. RMSE for Pittsburgh compound B (PiB) distribution volume ratio (DVR) was 0.063 DVR. SILA-estimated p-tau217 concentration residuals were similarly distributed for shorter (~3 years) and longer (~7 years) follow-up times and for low vs. high concentrations (**Supplemental Figure 3**)

SILA estimated p-tau217+ ages in subsets of Lilly and ALZpath converters

We observed 27 participants convert from Lilly p-tau217 negative to positive and 23 participants convert from ALZpath p-tau217 negative to positive. **Supplemental Figure 4** shows the observed p-tau217 concentrations for each of the convertors plotted as a function of the estimated p-tau217+ time, along with boxplots indicating the difference between the “observed” conversion age and the SILA-estimated p-tau217+ age. The mean (95% CI) of the difference between observed and SILA-estimate p-tau217+ age was –0.95 (-1.77, -0.13) years for the Lilly assay and –0.58 (-1.87, 0.71) years for the ALZpath assay. T-tests of this difference indicated the Lilly estimated p-tau217+ age was significantly different from zero (p = 0.02) whereas this difference was not significantly different from zero for the ALZpath assay (p = 0.36).

Comparisons of estimated p-tau217+ ages

Paired comparisons of estimated p-tau217+ ages were more variable in the p-tau217- participants compared to the p-tau217+ participants, as was expected. Amongst the p-tau217+ participants, the mean difference (95% CI) between Alzpath and Lilly estimated p-tau217+ ages among p-tau217+ participants was 2.40 (1.25, 3.54) years with estimated Alzpath p-tau217+ occurring at an older age on average than Lilly p-tau217+. At the person-level, differences ranged from -5.36 to 18.62 years (**Supplemental Figure 5**). Differences in estimated p-tau217+ ages corresponded to differences in observed concentrations between Alzpath and Lilly assays at the reference observation used to derive time estimates for each participant (**Supplemental Figure 6)**.

**Supplemental Figure 1**

A B C


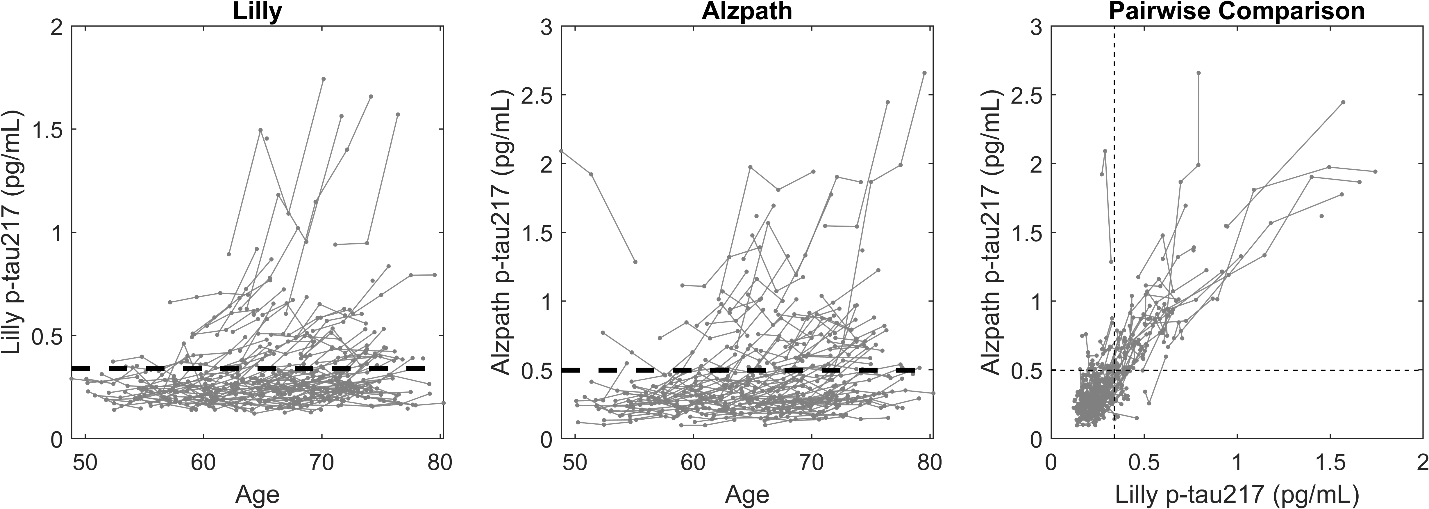


**Supplemental Figure 1.** Observed Lilly MSD (A) and Quanterix Alzpath (B) p-tau217 data vs. age, and vs. each other (C) for person- and time-matched plasma samples. Dashed lines indicate positivity thresholds for each assay derived in the same subset of Lilly p-tau217 non-accumulators. Each gray line represents longitudinal observations within an individual (N, matched subjects = 172; N, plasma observations = 520).

**Supplemental Figure 2**

A B


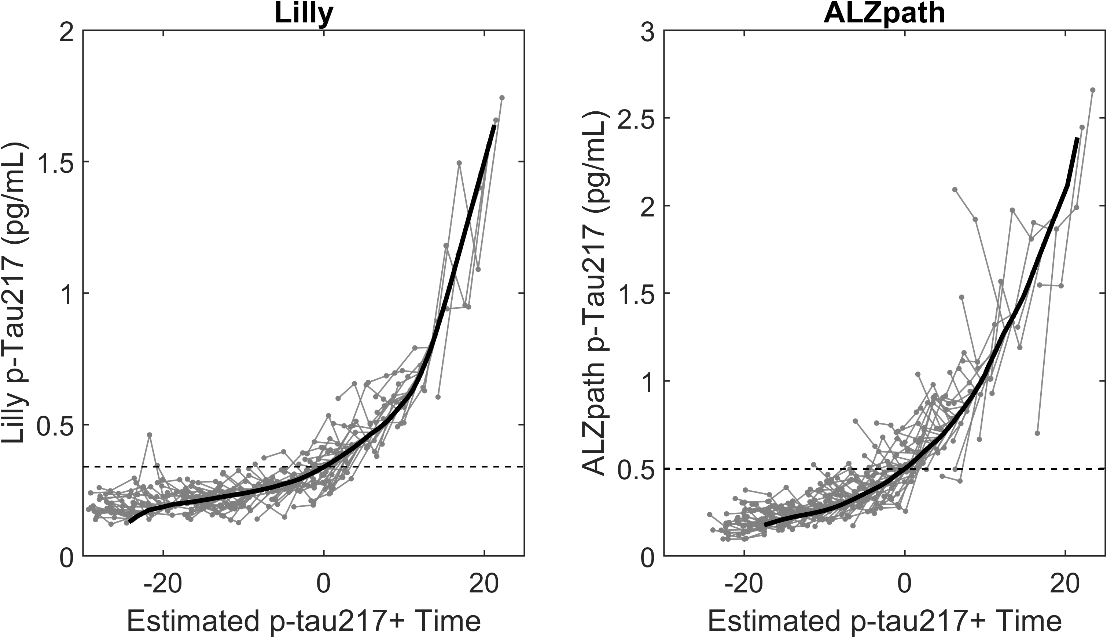


**Supplemental Figure 2.** SILA-modeled p-tau217 accumulation trajectories for Lilly MSD (A) and Quanterix Alzpath (B) plasma p-tau217. Gray lines represent longitudinal observations within individual participants (N, matched subjects = 172; N, plasma observations = 520). Thick black lines are the discrete SILA modeled p-tau217 concentration vs. estimated p-tau217+ time. Dashed black line indicates the threshold for positivity.

**Supplemental Figure 3**

**
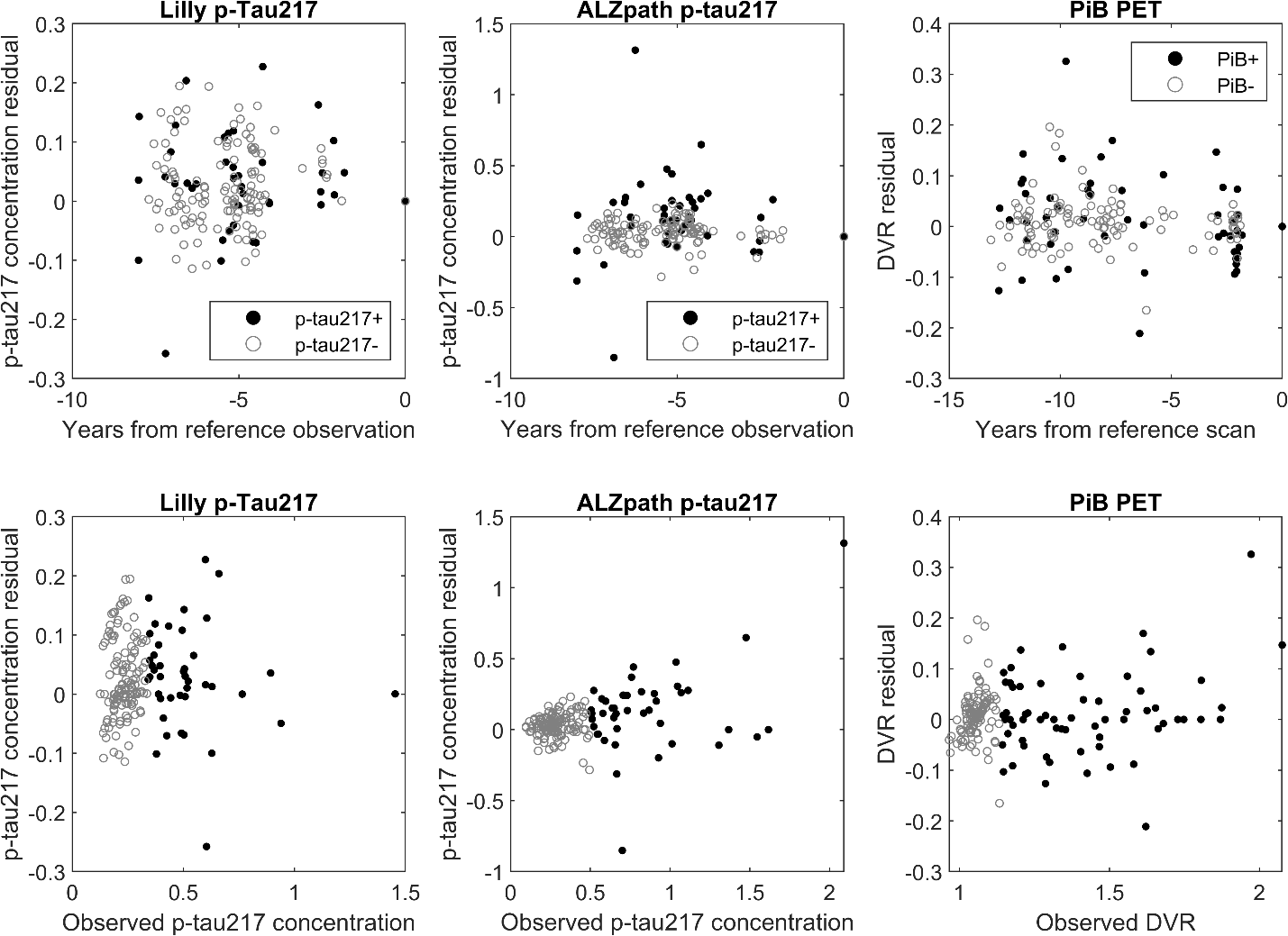
**

**Supplemental Figure 3.** SILA model residuals for estimated values at earlier timepoints plotted as a function for years from reference observation (top row) and observed value (bottom row) for Lilly MSD plasma p-tau217 (left), ALZpath plasma p-tau217 (middle), and PiB DVR (right; shown for context to prior amyloid PET validation).

**Supplemental Figure 4**

**A B C**


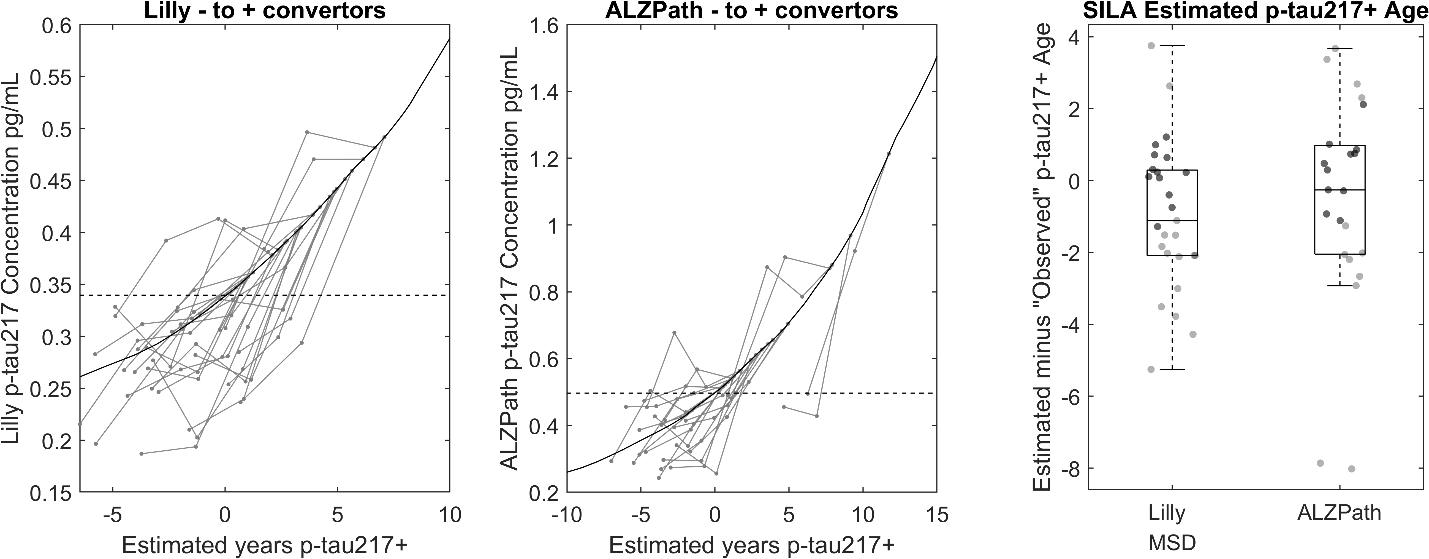


**Supplemental Figure 4.** Observed p-tau217 concentrations for Lilly (A) and ALZpath (B) assays vs. estimated years p-tau217+ in subsets of participants observed to convert from p-tau217 negative to positive. Panel (C) shows the difference between the SILA-estimated and observed p-tau217 positive ages. The “observed” p-tau217+ age is calculated as the age midpoint between the last p-tau217 negative and first p-tau217 positive observations and were calculated separately for each assay. T-tests of this difference indicated the Lilly estimated p-tau217+ age was significantly different from zero (p = 0.02) whereas this difference was not significantly different from zero for the ALZpath assay (p = 0.36).

**Supplemental Figure 5**

A B C


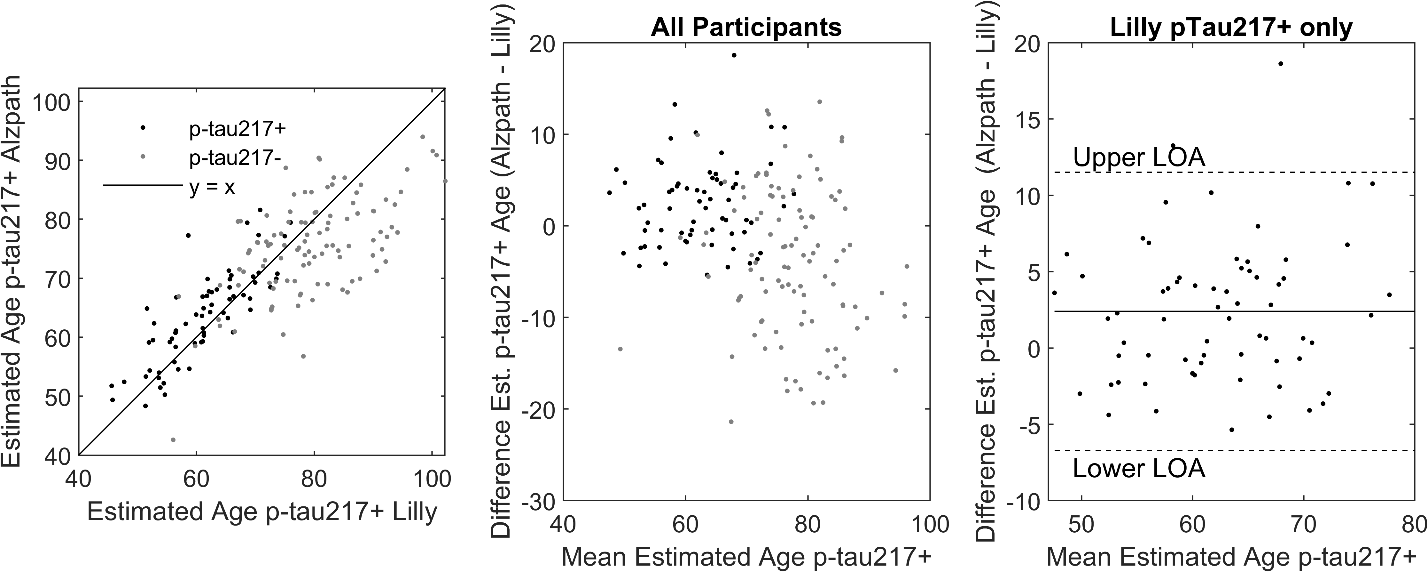


**Supplemental Figure 5.** Comparisons of estimated p-tau217+ ages derived from Alzpath and Lilly assays (A) with Bland-Altman plots showing the difference between SILA-estimated p-tau217 ages for all participants (n=172) (B) and for the subset (n=56) that were observed to be Lilly p-tau217+ (C). Dots indicate individual participants (N, matched subjects = 172). P-tau217 positivity is indicated by color (red = p-tau217+; black = p-tau217-). Note that estimated p-tau217+ ages for p-tau217- individuals are censored in the primary analyses but are shown here for sake of completeness. In panel (C), the upper and lower limits of agreement (LOA) are shown as dashed black lines and were calculated as ± 1.96 standard deviations (SD) of the mean.

**Supplemental Figure 6**


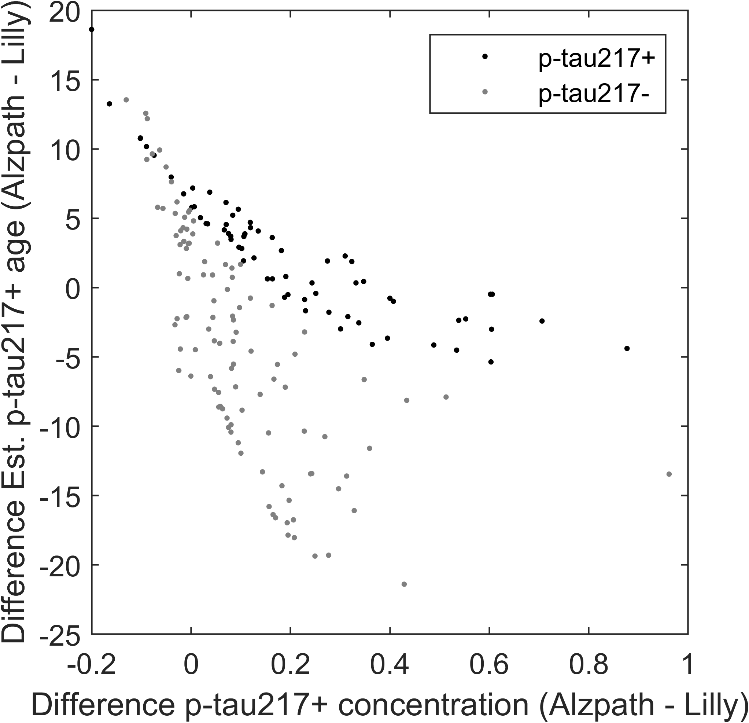


**Supplemental Figure 6.** The difference in estimated p-tau217+ ages vs. the difference in p-tau217 concentrations between Alzpath and Lilly assays (N, matched subjects = 172). Black points indicate participants that were p-tau217+ on the Lilly assay, whereas gray points are Lilly p-tau217- participants.

**Supplemental Tables**

| **Supplemental Table 1. Tau PET subsample characteristics** | | | | |
| --- | --- | --- | --- | --- |
|  |  | **Clinical diagnosis at tau PET scan** | | |
| **Characteristic** | **Overall**  N = 148 | **CU**  N = 130 | **MCI**  N = 14 | **Dementia**  N = 4 |
| **Age at tau PET (years)** | 70.27 (6.19) | 69.89 (6.18) | 72.32 (5.71) | 75.21 (6.01) |
| **Tau PET follow-up (years)** | 1.72 (1.41) | 1.72 (1.39) | 1.45 (1.50) | 2.46 (1.73) |
| **Number of tau PET scans** | 1.74 (0.64) | 1.73 (0.62) | 1.71 (0.73) | 2.25 (0.96) |
| **Amyloid and Lilly ptau217 positivity** |  |  |  |  |
| A-ptau217- | 67 (45%) | 67 (52%) | 0 (0%) | 0 (0%) |
| A+ptau217- | 25 (17%) | 23 (18%) | 2 (14%) | 0 (0%) |
| A-ptau217+ | 2 (1.4%) | 2 (1.5%) | 0 (0%) | 0 (0%) |
| A+ptau217+ | 54 (36%) | 38 (29%) | 12 (86%) | 4 (100%) |
| **Amyloid and Alzpath Quanterix ptau217 positivity** |  |  |  |  |
| A-ptau217- | 61 (41%) | 61 (47%) | 0 (0%) | 0 (0%) |
| A+ptau217- | 32 (22%) | 29 (22%) | 3 (21%) | 0 (0%) |
| A-ptau217+ | 8 (5.4%) | 8 (6.2%) | 0 (0%) | 0 (0%) |
| A+ptau217+ | 47 (32%) | 32 (25%) | 11 (79%) | 4 (100%) |
| **EC tau positivity** | 32 (22%) | 22 (17%) | 6 (43%) | 4 (100%) |
| **ITG tau positivity** | 19 (13%) | 9 (6.9%) | 6 (43%) | 4 (100%) |
| **Female** | 97 (66%) | 83 (64%) | 10 (71%) | 4 (100%) |
| **Education** |  |  |  |  |
| BA | 107 (72%) | 93 (72%) | 12 (86%) | 2 (50%) |
| No BA | 41 (28%) | 37 (28%) | 2 (14%) | 2 (50%) |
| ***APOE* e4 carriers** | 73 (49%) | 59 (45%) | 11 (79%) | 3 (75%) |
| Values shown as Mean (SD) or n (%)  Abbreviations: A+/-, amyloid positivity; APOE, apolipoprotein; BA, bachelor’s degree; CU, cognitively unimpaired; EC, Entorhinal cortex; ITG, Inferior temporal gyrus; MCI, mild cognitive impairment | | | | |

**Supplemental Table 2. Tau PET accumulation in A+ Lilly ptau217+ individuals as a function of age, amyloid time, and Lilly p-tau217 time**

| **Comparison of models fit in N=54 A+ Lilly p-tau217+ individuals with n=103 tau PET observations** | **AIC** | **Marginal R^2^** |
| --- | --- | --- |
| **Entorhinal (EC) tau** | | |
| EC MK-6240 SUVR ~ **Age at tau PET** + (1 \| participant) | 67.5 | 0.13 |
| EC MK-6240 SUVR ~ **A+ time at tau PET** + (1 \| participant) | 41.3^a^ | 0.41 |
| EC MK-6240 SUVR ~ **p-tau217+ time at tau PET** + (1 \| participant) | 23.7^a,b^ | 0.48 |
| **ITG tau** | | |
| ITG MK-6240 SUVR ~ **Age at tau PET** + (1 \| participant) | 120.9 | 0.08 |
| ITG MK-6240 SUVR ~ **A+ time at tau PET** + (1 \| participant) | 100.5^c^ | 0.32 |
| ITG MK-6240 SUVR ~ **p-tau217+ time at tau PET** + (1 \| participant) | 82.2^c,d^ | 0.42 |

Each model included 103 tau PET observations for 54 A+ptau217+ individuals with available tau PET data. Age, A+ time, and p-tau217+ time at tau PET were each mean centered and significant (*p*<0.001) predictors in all models.

^a^Likelihood ratio tests indicated A+ time and p-tau217+ time explained significantly (*P*<.001) more variance in EC MK-6240 tau standardized uptake value ratio (SUVR) compared to age at tau PET

^b^Likelihood ratio tests indicated p-tau217+ time explained significantly (*P*<.001) more variance in EC MK-6240 tau SUVR compared to A+ time at tau PET

^c^Likelihood ratio tests indicated A+ time and p-tau217+ time explained significantly (*P*<.001) more variance in ITG MK-6240 tau SUVR compared to age at tau PET

^d^Likelihood ratio tests indicated p-tau217+ time explained significantly (*P*<.001) more variance in ITG MK-6240 tau SUVR compared to A+ time at tau PET

**Supplemental Table 3. Tau PET accumulation in A+ Alzpath Quanterix ptau217+ individuals as a function of age, amyloid time, and Alzpath Quanterix p-tau217 time**

| **Comparison of models fit in N=47 A+ Alzpath Quanterix p-tau217+ individuals with n=83 tau PET observations** | **AIC** | **Marginal R^2^** |
| --- | --- | --- |
| **Entorhinal (EC) tau** | | |
| EC MK-6240 SUVR ~ **Age at tau PET** + (1 \| participant) | 57.0 | 0.07 |
| EC MK-6240 SUVR ~ **A+ time at tau PET** + (1 \| participant) | 32.4^a^ | 0.38 |
| EC MK-6240 SUVR ~ **p-tau217+ time at tau PET** + (1 \| participant) | 33.0^a^ | 0.33 |
| **ITG tau** | | |
| ITG MK-6240 SUVR ~ **Age at tau PET**+ (1 \| participant) | 107.7 | 0.06 |
| ITG MK-6240 SUVR ~ **A+ time at tau PET**+ (1 \| participant) | 89.2^b^ | 0.31 |
| ITG MK-6240 SUVR ~ **p-tau217+ time at tau PET** + (1 \| participant) | 89.7^b^ | 0.26 |

Each model included 83 tau PET observations for 47 A+ptau217+ individuals with available tau PET data. Age, A+ time, and Alzpath p-tau217+ time at tau PET were each mean centered and significant (*p*<0.05) predictors in all models.

^a^Likelihood ratio tests indicated A+ time and p-tau217+ time explained significantly (*P*<.001) more variance in EC MK-6240 tau SUVR compared to age at tau PET, but were not significantly different from one another

^b^Likelihood ratio tests indicated A+ time and p-tau217+ time explained significantly (*P*<.001) more variance in ITG MK-6240 tau SUVR compared to age at tau PET, but were not significantly different from one another

**Supplemental Table 4. PACC-3 decline in A+ptau217+ individuals as a function of age, amyloid time, and Alzpath p-tau217 time**

| **Comparison of models fit in N=54 A+p-tau217+ individuals with n=308 PACC-3 observations** | ∆**AICc** | **Marginal R^2^** |
| --- | --- | --- |
| zPACC3 ~ **Age at PACC3^2^** + covariates + (age\|participant) | 19.4 | 0.29 |
| zPACC3 ~ **A+ time^2^** + covariates + (A+ time\|participant) | 28.5 | 0.40 |
| zPACC3 ~ **p-tau217+ time^2^** + covariates + (p-tau217+ time \| participant) | 0 | 0.43 |

Each model included 274 zPACC-3 observations for 54 A+ptau217+ individuals. Each model included one of the following time-varying predictors of interest: linear and quadratic age at PACC-3, linear and quadratic p-tau217+ time at PACC-3, or linear and quadratic A+ time at PACC-3. All models covaried for age at baseline PACC-3, sex, and the number of prior exposures to the cognitive battery (i.e., practice effects). All models included lower-order terms. Age, A+ time, and Alzpath p-tau217+ time were each mean centered and significant (*p*<0.001) quadratic and linear predictors in each model. Model fits were compared using corrected Akaike information criteria (AIC).

**Supplemental Figures**

**Supplemental Figure 7. Temporal modeling of amyloid PET and Lilly p-tau217 in 173 individuals from WRAP, including Lilly p-tau217 outlier**

**
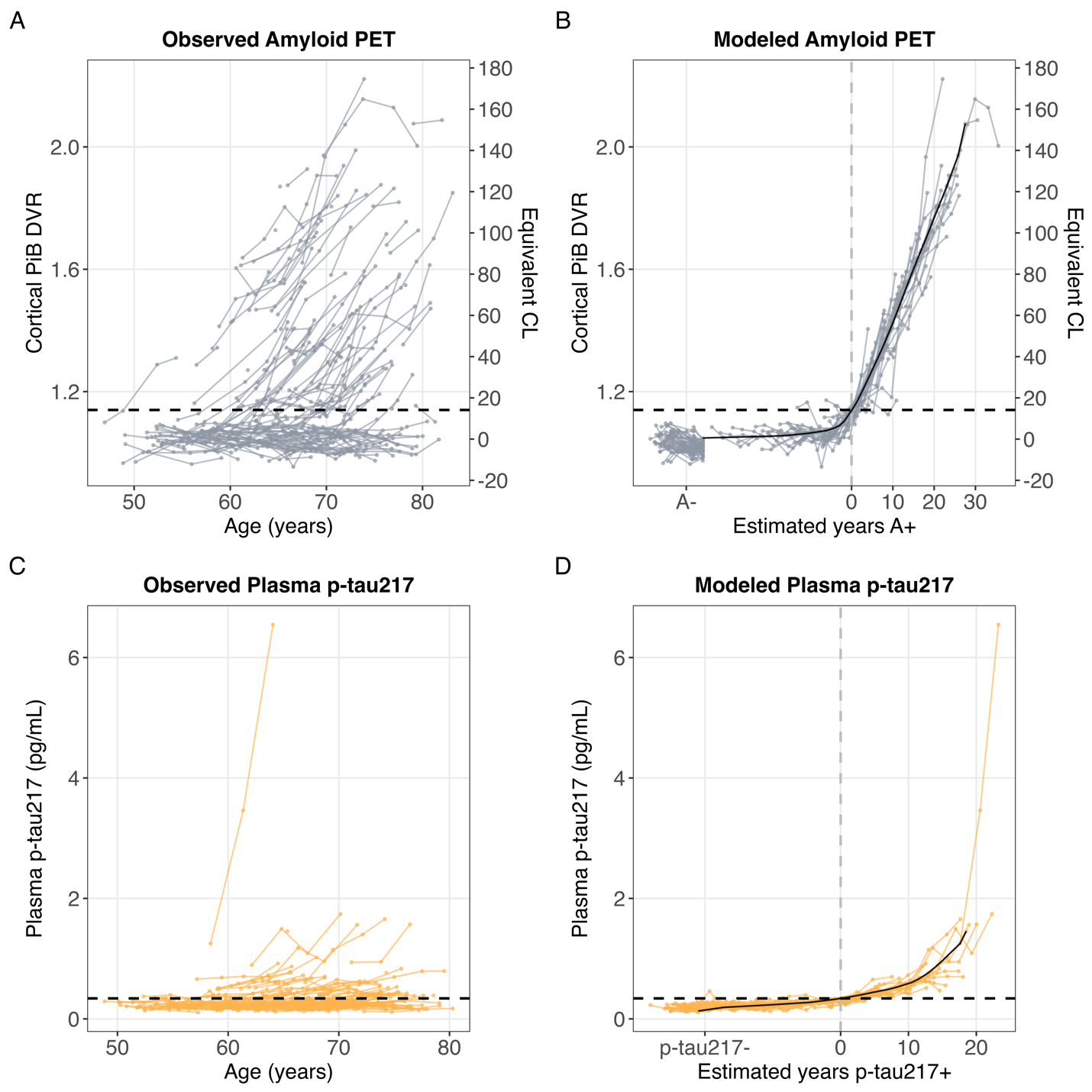
**

**Supplemental Figure 7. Temporal modeling of amyloid PET and Lilly p-tau217 in 173 individuals from WRAP, including Lilly p-tau217 outlier.** Observed cortical PiB DVR **(A)** and plasma p-tau217 **(C)** as a function of age and the resultant PiB **(B)** and p-tau217 **(D)** accumulation trajectories from the SILA model (solid black lines show integrated curve from SILA). Zero years for modeled amyloid PET and plasma p-tau217 timelines (gray vertical dashed lines) were defined as the point the model intersected the positivity threshold (black horizontal dashed line) for each biomarker (PiB DVR = 1.14 (14.1 equivalent Centiloids (CL)); p-tau217 = 0.34 pg/mL).

**Supplemental Figure 8. Amyloid PET onset generally precedes Lilly plasma p-tau217 onset** **(including Lilly p-tau217 outlier)**
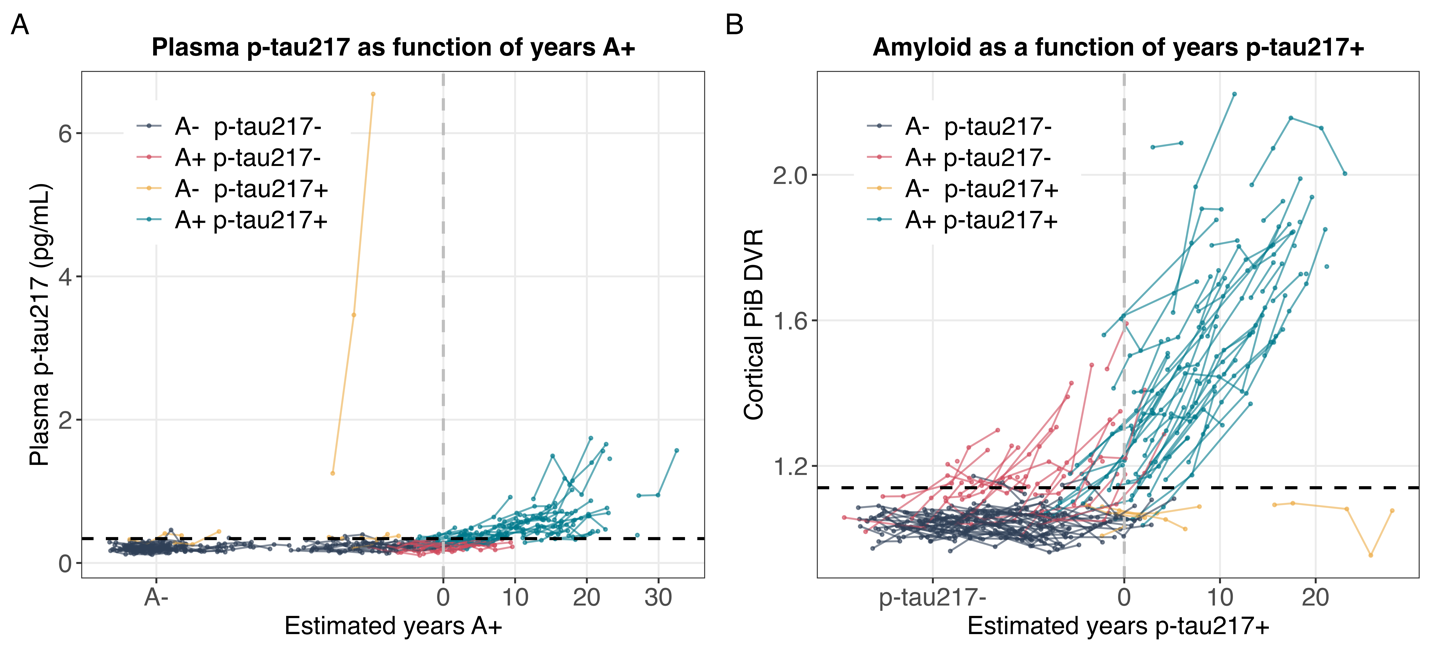
 **Supplemental Figure 8. Amyloid PET onset generally precedes Lilly plasma p-tau217 onset** **(including Lilly p-tau217 outlier).** Participants were classified as biomarker positive or negative based on their last observed biomarker assessment, which resulted in four biomarker groups: A- p-tau217- (n=79; navy), A+ p-tau217- (n=27; pink), A- p-tau217+ (n=6; yellow), A+ p-tau217+ (n=61; blue). (**A**) Observed plasma p-tau217 as a function of estimated years A+ demonstrates that most individuals become p-tau217+ (i.e., crossing the horizontal black dashed positivity threshold) after amyloid onset (i.e., years A+=0; Gray vertical dashed line). (**B**) Observed cortical PiB DVR as a function of estimated years p-tau217+ demonstrates that many individuals become A+ (i.e., crossing the horizontal black dashed positivity threshold) before p-tau217 onset (i.e., years p-tau217+=0; Gray vertical dashed line).

**Supplemental Figure 9. Amyloid PET onset generally precedes Alzpath plasma p-tau217 onset**


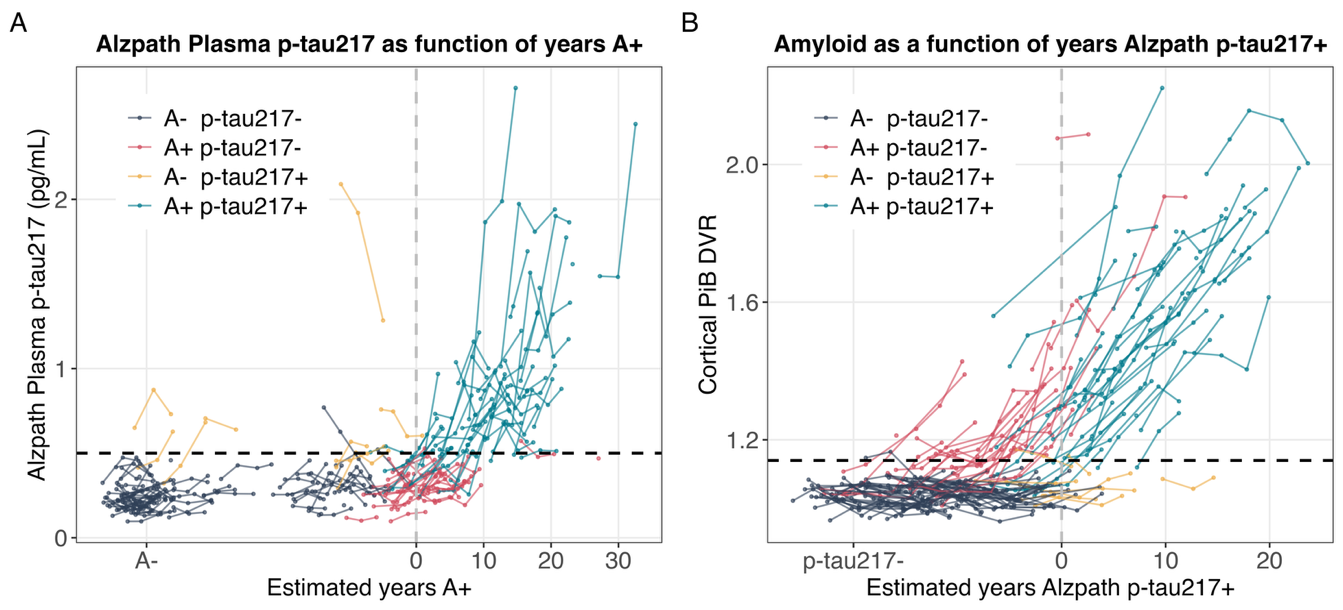


**Supplemental Figure 9. Amyloid PET onset generally precedes Alzpath** **plasma p-tau217 onset**. Participants were classified as biomarker positive or negative based on their last observed biomarker assessment, which resulted in four biomarker groups: A- Alzpath p-tau217- (n=75; navy), A+ Alzpath p-tau217- (n=34; pink), A- Alzpath p-tau217+ (n=9; yellow), A+ Alzpath p-tau217+ (n=54; blue). (A) Observed plasma p-tau217 as a function of estimated years A+ demonstrates that most individuals become p-tau217+ (i.e., crossing the horizontal black dashed positivity threshold) after amyloid onset (i.e., years A+=0; Gray vertical dashed line). (B) Observed cortical PiB DVR as a function of estimated years p-tau217+ demonstrates that many individuals become A+ (i.e., crossing the horizontal black dashed positivity threshold) before p-tau217 onset (i.e., years p-tau217+=0; Gray vertical dashed line).

**Supplemental Figure 10.**


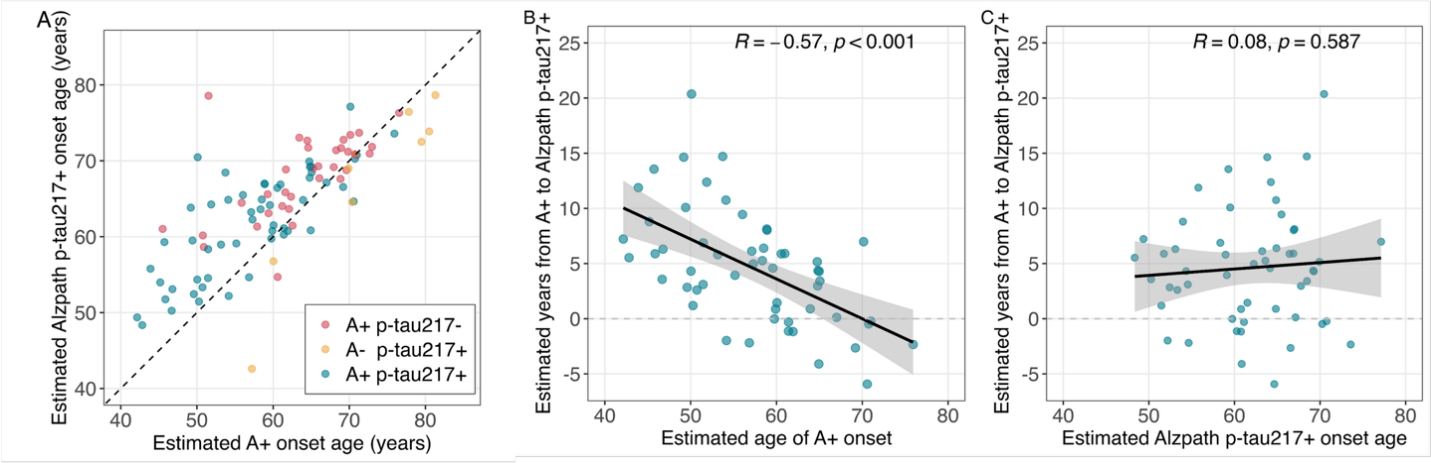


**Supplemental Figure 10. Comparison of amyloid PET and plasma Alzpath p-tau217 onset ages**. A comparison of biomarker onset ages (A) for individuals that became biomarker positive on at least one biomarker (e.g., A+ p-tau217-, n=34 (pink); A- p-tau217+, n=9 (yellow); and A+ p-tau217+, n=54 total (teal). Individuals above the dashed diagonal line had an A+ onset age that preceded their p-tau217+ onset age. The estimated time difference between A+ onset and p-tau217+ onset is shown as a function of A+ onset age (B) and p-tau217+ onset age (C) for the group that was observed to be positive on both biomarkers (e.g., A+ p-tau217+, teal; n=54). In panels B and C, Pearson’s R and corresponding p value is shown for the relationship between time from A+ to p-tau217+ and estimated A+ onset age (B) and estimated p-tau217 onset age (C). In this A+ p-tau217+ subset, A+ onset generally preceded p-tau217+ by an average(SD) 4.6 (5.2) years, and younger estimated age at A+ onset was associated with longer time to p-tau217+.

**Supplemental Figure 11. Observed Tau PET SUVR as a function of biomarker time with censored observations (Lilly p-tau)**
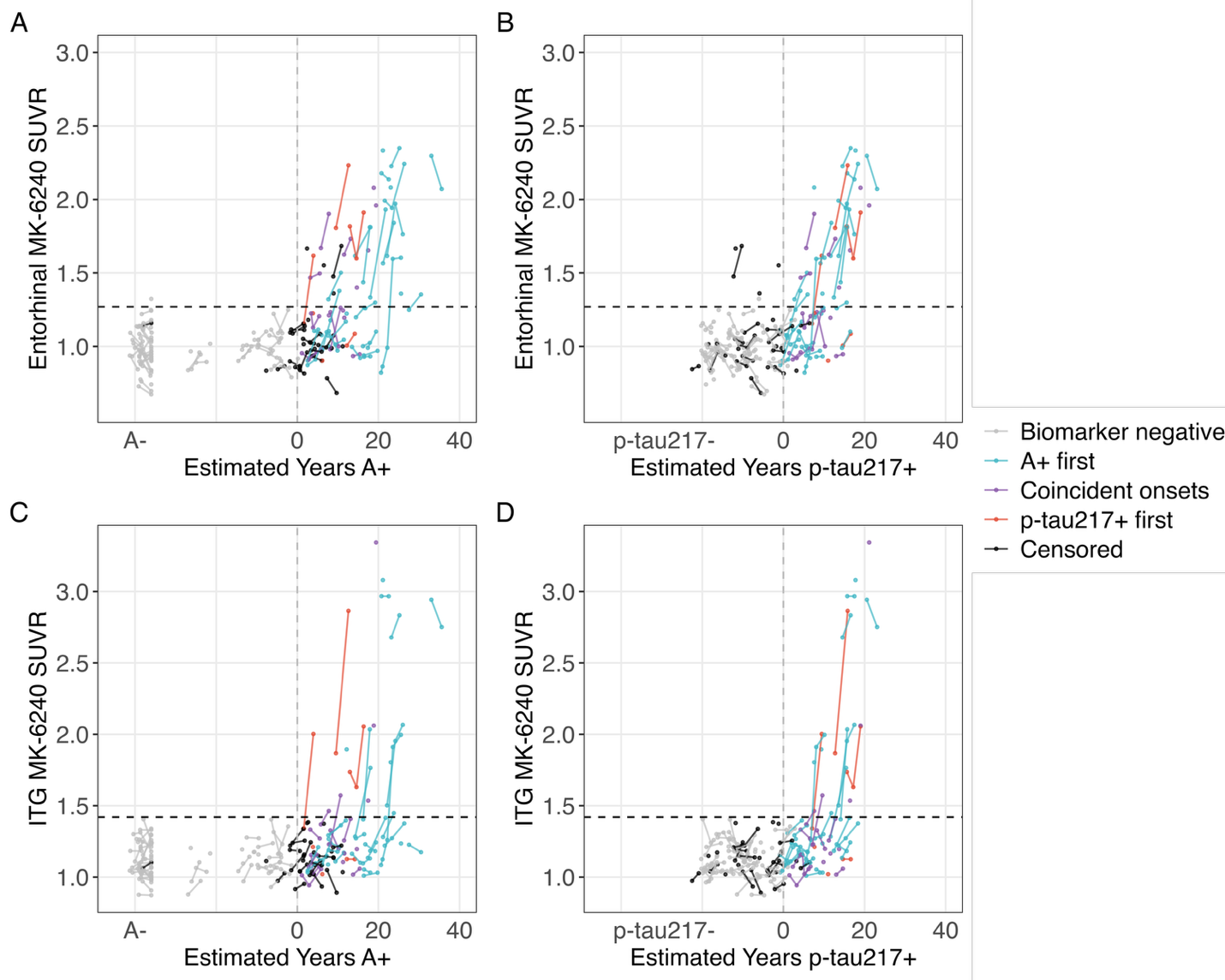


**Supplemental Figure 11. Observed Tau PET SUVR as a function of biomarker time with censored observations.** Observed entorhinal cortex (A-B) and inferior temporal gyrus (ITG; C-D) MK-6240 tau PET SUVR plotted as a function of estimated years A+ (A)(C) and estimated years Lilly p-tau217+ (B)(D) for all study 148 participants (i.e., including those “censored” from Figure 4 due to having only one positive biomarker). Lines represent longitudinal tau PET observations (connected dots) within individual participants (n=148 participants; n=258 tau PET observations).The horizontal black dashed lines indicate regional tau PET positivity thresholds, vertical gray dashed lines indicate amyloid onset (i.e. Years A+ =0) and Lilly p-tau217+ onset (i.e. Years p-tau217+ =0), and the colors indicate relative biomarker timing: Biomarker negative (gray, n=67), A+ first (A+ onset age > 2 years before p-tau217+ onset age; light blue, n=32), Coincident onsets (A+ and p-tau217+ onsets within 2 years; purple, n=16), p-tau217+ first (p-tau217+ onset age > 2 years before A+ onset age; red, n =6), and censored cases (e.g. participants with only one positive biomarker, so relative timing could not be inferred; black, n=27).

**Supplemental Figure 12.** Observed Tau PET SUVR as a function of biomarker time (Alzpath p-tau217) **
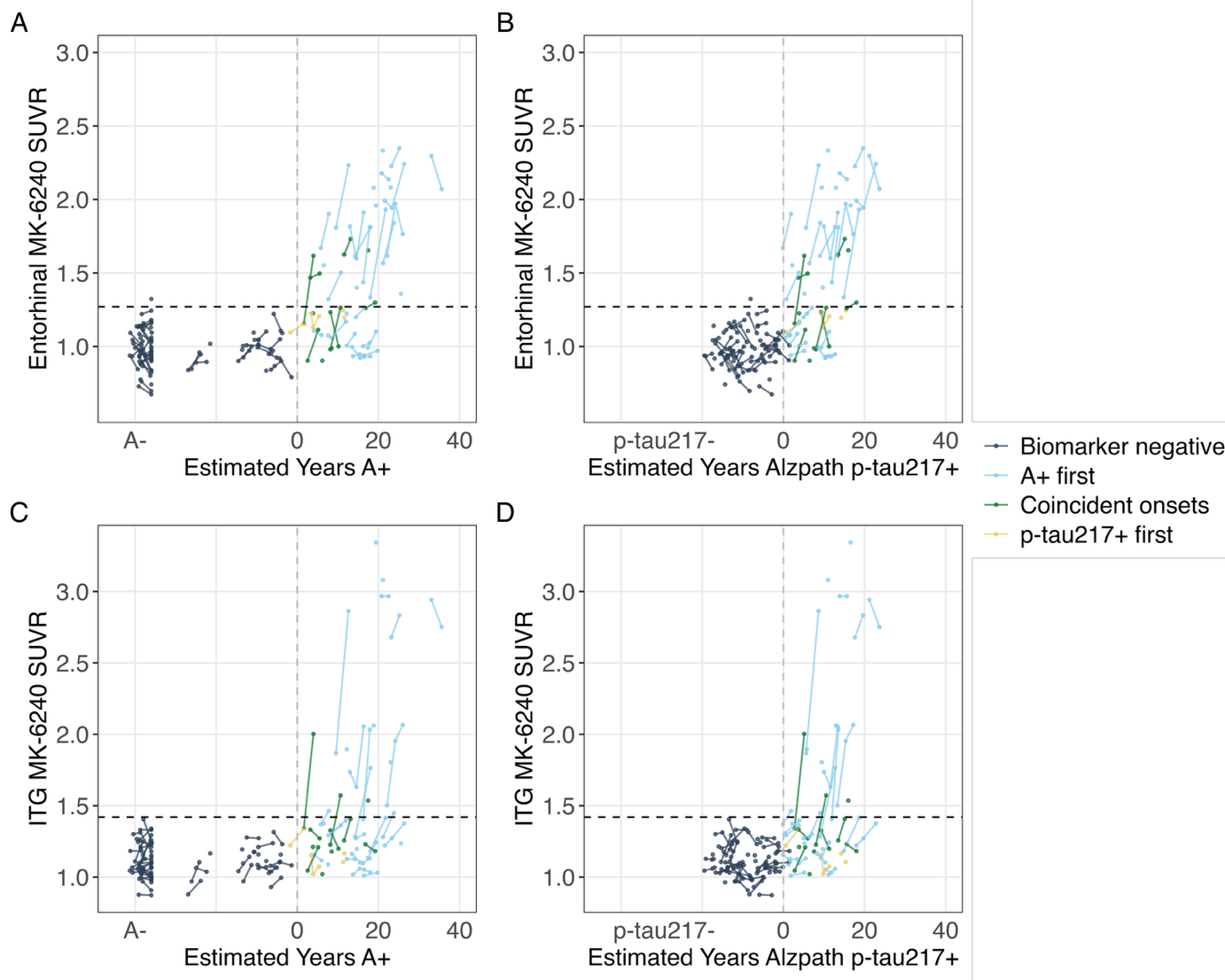
**

**Supplemental Figure 12. Observed Tau PET SUVR as a function of biomarker time (Alzpath p-tau217).** Observed entorhinal cortex (A-B) and inferior temporal gyrus (ITG; C-D) MK-6240 tau PET standardized uptake value ratio (SUVR) plotted as a function of estimated years A+ (A,C) and estimated years Alzpath p-tau217+ (B,D) for participants observed to be A- p-tau217- (n=61 participants) or A+ p-tau217+ (n=47 participants) at their last biomarker observations who had available tau PET imaging. Lines represent longitudinal tau PET observations (connected dots) within individual participants (n=108 participants; n=186 tau PET observations).The horizontal black dashed lines indicate regional tau PET positivity thresholds, vertical gray dashed lines indicate amyloid onset (i.e. Years A+ =0) and Alzpath p-tau217+ onset (i.e. Years p-tau217+ =0), and the colors indicate relative biomarker timing: Biomarker negative (navy, n=61), A+ first (A+ onset age > 2 years before p-tau217+ onset age; light blue, n=32), Coincident onsets (A+ and p-tau217+ onsets within 2 years; green, n=11), and p-tau217+ first (p-tau217+ onset age > 2 years before A+ onset age; yellow, n =4).

**Supplemental Figure 13.** Observed Tau PET SUVR as a function of biomarker time with censored observations (Alzpath p-tau217)


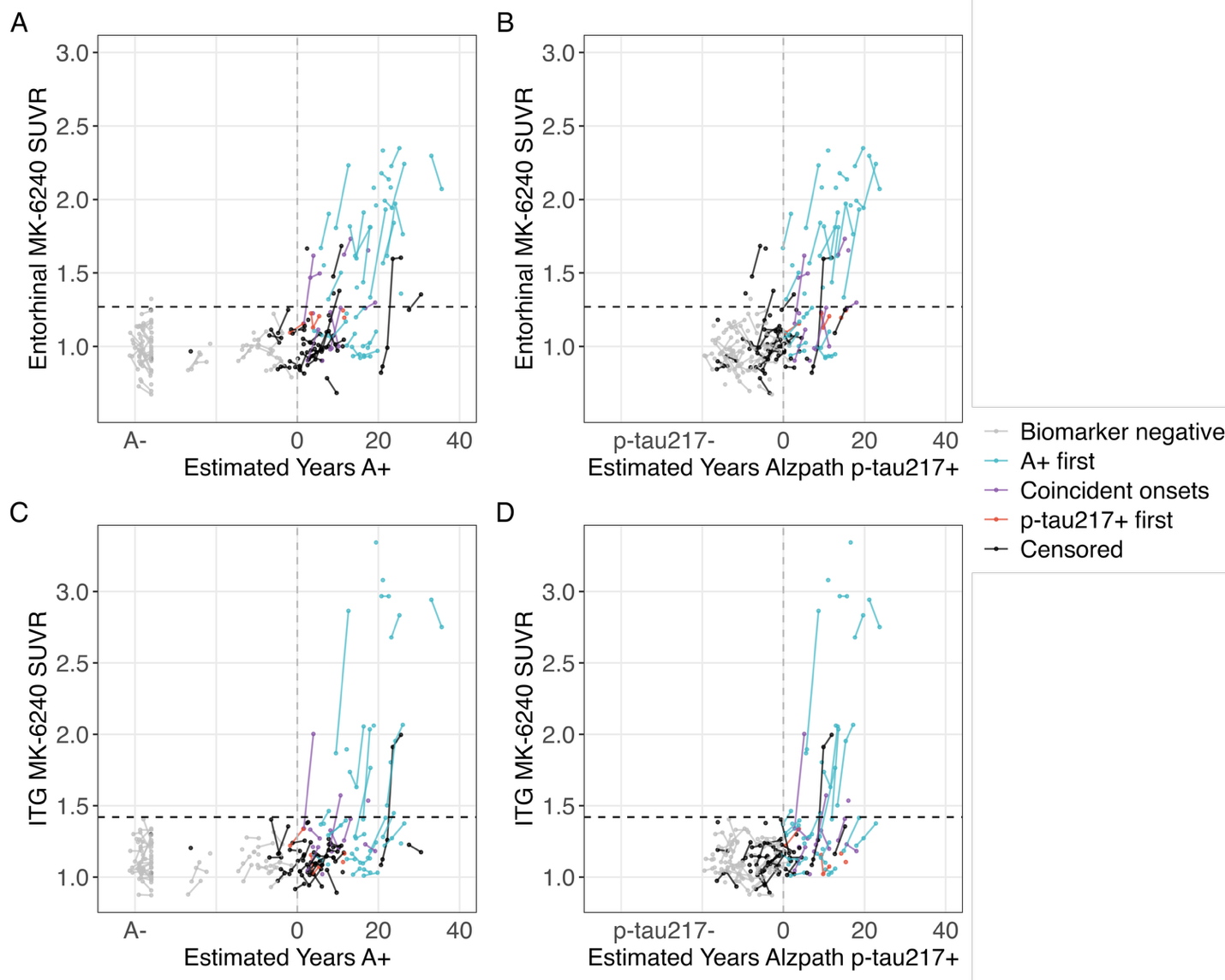


**Supplemental Figure 13. Observed Tau PET SUVR as a function of biomarker time with censored observations (Alzpath p-tau217).** Observed entorhinal cortex (A-B) and inferior temporal gyrus (ITG; C-D) MK-6240 tau PET SUVR plotted as a function of estimated years A+ (A)(C) and estimated years p-tau217+ (B)(D) for all study 148 participants (i.e., including those “censored” from Supplemental Figure 7 due to having only one positive biomarker). Lines represent longitudinal tau PET observations (connected dots) within individual participants (n=148 participants; n=258 tau PET observations).The horizontal black dashed lines indicate regional tau PET positivity thresholds, vertical gray dashed lines indicate amyloid onset (i.e. Years A+ =0) and p-tau217+ onset (i.e. Years p-tau217+ =0), and the colors indicate relative biomarker timing: Biomarker negative (gray, n=61), A+ first (A+ onset age > 2 years before p-tau217+ onset age; light blue, n=32), Coincident onsets (A+ and p-tau217+ onsets within 2 years; purple, n=11), p-tau217+ first (p-tau217+ onset age > 2 years before A+ onset age; red, n =4), and censored cases (e.g. participants with only one positive biomarker, so relative timing could not be inferred; black, n=40).

**Supplemental Figure 14.** Relative timing of biomarker positivity as a function of amyloid and Alzpath p-tau217 time.

A


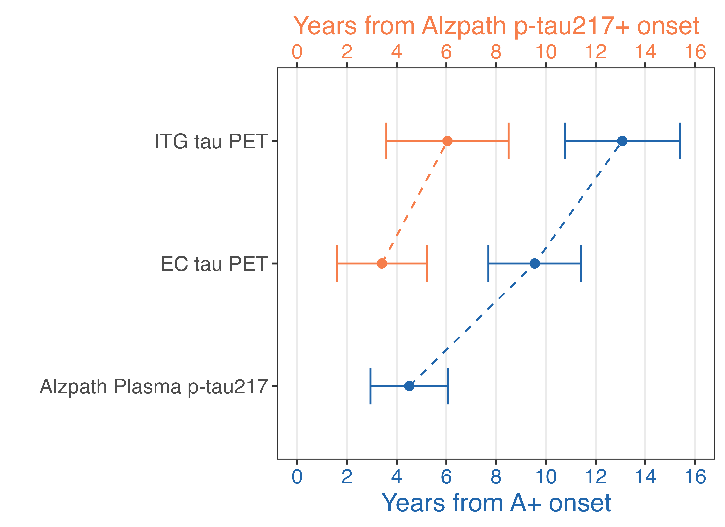


B


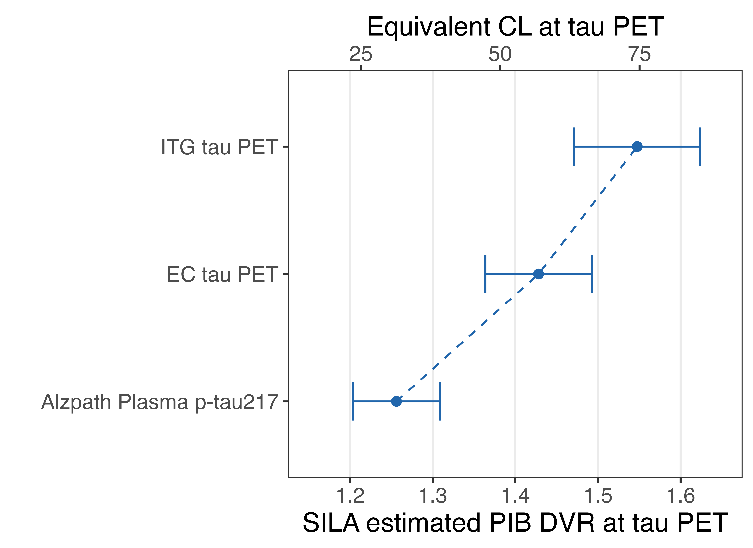


C


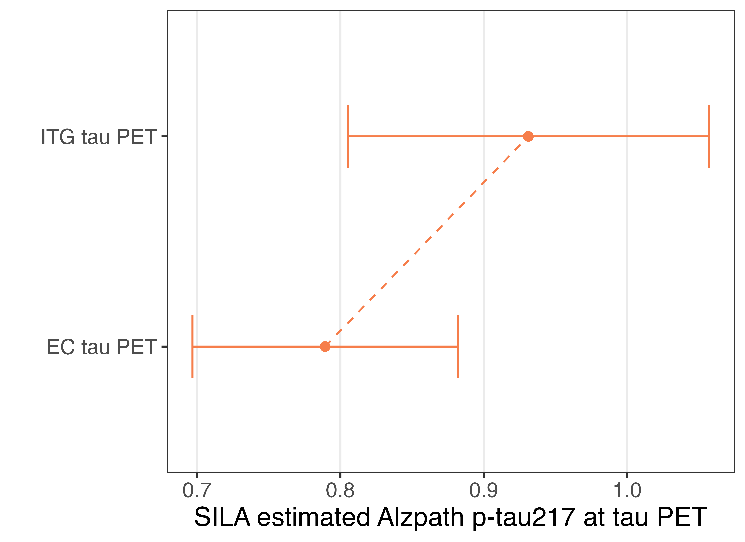


**Supplemental Figure 14. Relative timing of biomarker positivity as a function of amyloid and Alzpath p-tau217 time.** Estimated time to Alzpath plasma p-tau217 and regional tau positivity from amyloid PET (A+) onset among A+ individuals (n, participants=79; n, tau PET observations=143). Linear regressions of A+ time predicting longitudinal plasma p-tau217 at the time of the tau PET scan, entorhinal (EC) and inferior temporal gyral (ITG) tau PET as well as linear regression of Alzpath plasma p-tau217+ time predicting EC and ITG tau PET were conducted. We then used inverse regression (Wald interval, delta method) to predict time from A+ to plasma p-tau217, EC tau PET, and ITG tau PET positivity as well as time from plasma p-tau217+ to EC and ITG tau PET positivity. Panel A shows time estimates (dots) of biomarker positivity along the amyloid timeline in blue and time estimates of tau PET positivity along the p-tau217 timeline shown in orange, with error bars representing the standard error for each time estimate. On average, the magnitude of tau biomarker changes along the amyloid timeline followed the expected pattern of tau accumulation, such that detectable changes in plasma p-tau217 occurred before detectable changes in EC tau PET accumulation which preceded detectable tau spread outside of the medial temporal lobe (ITG tau PET accumulation). For additional context, we also ran models using amyloid PET DVR/CL and Lilly p-tau217 concentration instead of time estimates, which show the estimated amyloid DVR and p-tau217 concentrations corresponding to plasma and tau PET positivity (panels B and C, respectively). Notably similar to our Lilly p-tau217 findings, we observed that among A+ individuals, on average Alzpath p-tau217+ occurred 4.5 (95% CI, 2.9-6.1) years after A+ onset with entorhinal tau positivity occurring 9.6 (95% CI, 7.7-11.4) years after A+ onset and inferior temporal gyrus tau positivity occurring 13.1 (95% CI, 10.8-15.4) years after A+ onset. Correspondingly, when we used SILA estimates of amyloid level rather than A+ time, we observed that on average, Alzpath plasma p-tau217+ occurred at approximately 32 CL (95% CI, 23-39 CL), entorhinal tau PET positivity occurred at 57 CL (95%CI, 47-66 CL) and inferior temporal gyrus T+ occurred at 75 CL (95%CI, 63-85 CL). In contrast, along the p-tau217 timeline, entorhinal tau PET positivity occurred on average 3.4 (95% CI,1.6-5.2) years after p-tau217+ onset and inferior temporal gyrus tau positivity occurred 6.0 (95% CI, 3.6-8.5) years after p-tau217+ onset among A+ individuals.

**Supplemental Figure 15.** Preclinical Alzheimer’s cognitive composite scores by age, A+ time, and Alzpath p-tau217+ time.


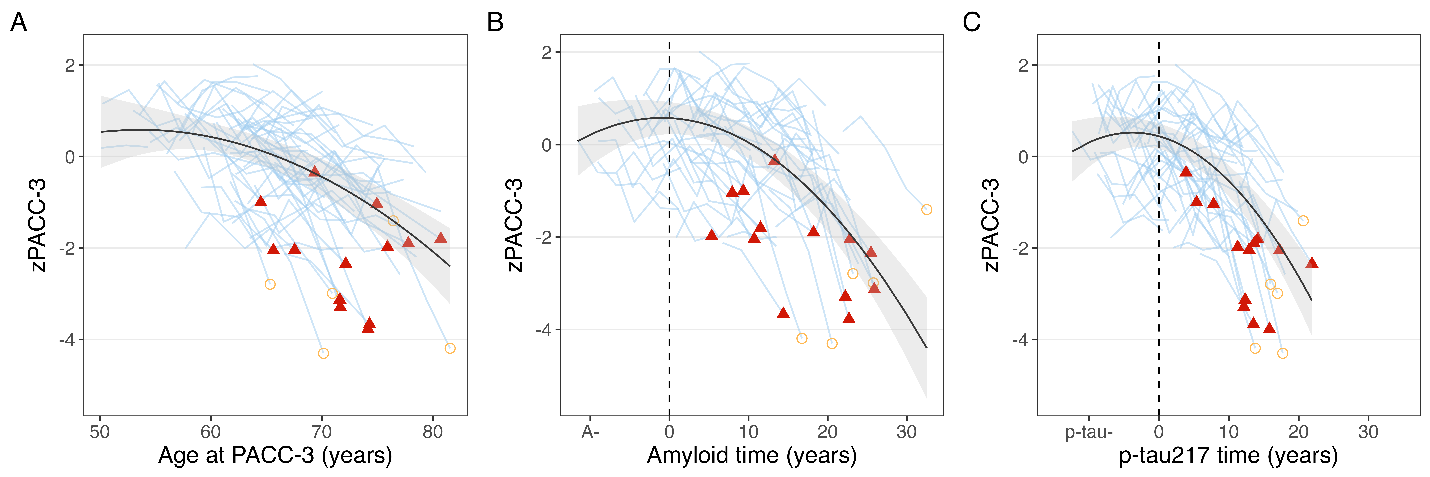

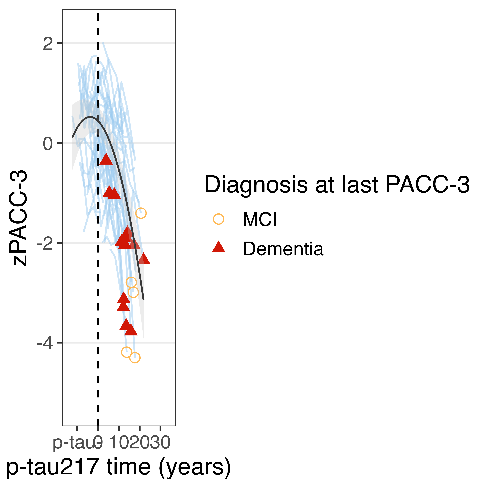


**Supplemental Figure 15. Preclinical Alzheimer’s cognitive composite scores by age, A+ time, and Alzpath p-tau217+ time.** Observed and modeled preclinical Alzheimer’s cognitive composite scores (zPACC-3) by age (**A**), A+ time (**B**), and Alzpath p-tau217+ time (**C**) for 54 A+p-tau217+ individuals with a total of 274 PACC-3 observations over an average 11.0 (3.27) years of follow-up. Light blue lines represent observed longitudinal PACC-3 trajectories for individual participants. All participants were cognitively unimpaired at baseline PACC-3 and their diagnosis at their most recent PACC-3 is indicated by shape (no shape = cognitively unimpaired; open yellow circle = mild cognitive impairment (MCI); filled red triangle = dementia). Dashed vertical lines indicate biomarker onset for amyloid (**B**) and p-tau217 (**C**), respectively. For each plot, zPACC-3 was modeled using mixed effects models including linear and quadratic time, baseline age at PACC-3, gender, practice, random intercept and random time slope where time was mean centered and operationalized as age at PACC-3 in panel (**A)**, A+ time at PACC-3 in panel (**B)**, and p-tau217+ time at PACC-3 in panel **C**. Modeled PACC-3 trajectories are shown as the black bold line and shaded areas indicate 95% CIs. Model comparisons are detailed in Supplemental Table 4.
